# Model of For3p-Mediated Actin Cable Assembly in Fission Yeast

**DOI:** 10.1371/journal.pone.0004078

**Published:** 2008-12-31

**Authors:** Hui Wang, Dimitrios Vavylonis

**Affiliations:** Department of Physics, Lehigh University, Bethlehem, Pennsylvania, United States of America; Wellcome Trust Sanger Institute, United Kingdom

## Abstract

Formin For3p nucleates actin cables at the tips of fission yeast cells for polarized cell growth. The results of prior experiments have suggested a possible mechanism for actin cable assembly that involves association of For3p near cell tips, For3p-mediated actin polymerization, retrograde flow of actin cables toward the cell center, For3p dissociation from cell tips, and cable disassembly. We used analytical and computational modeling to test the validity and implications of the proposed coupled For3p/actin mechanism. We compared the model to prior experiments quantitatively and generated predictions for the expected behavior of the actin cable system upon changes of parameter values. We found that the model generates stable steady states with realistic values of rate constants and actin and For3p concentrations. Comparison of our results to previous experiments monitoring the FRAP of For3p-3GFP and the response of actin cables to treatments with actin depolymerizing drugs provided further support for the model. We identified the set of parameter values that produces results in agreement with experimental observations. We discuss future experiments that will help test the model's predictions and eliminate other possible mechanisms. The results of the model suggest that flow of actin cables may establish actin and For3p concentration gradients in the cytoplasm that could be important in global cell patterning.

## Introduction

**Figure 1:**
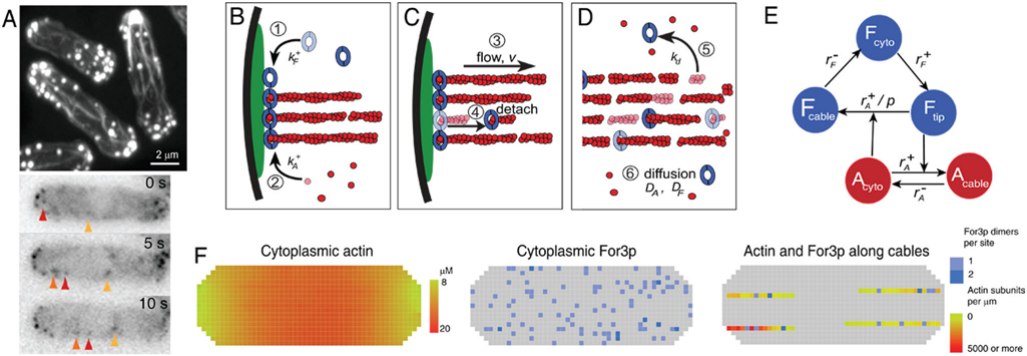
For3p-mediated actin cable dynamics in fission yeast. (A) Images of interphase yeast cells showing actin filaments labeled with phalloidin in fixed cells (top) and time-lapse images of cells expressing For3p-3GFP (bottom), from ref. [4] (reproduced with permission). Actin assembles into bundles (actin cables) and spots (actin patches). For3p localizes in cortical foci at cell tips from which it detaches and moves along actin cables (arrows). (B)–(D) Model of For3p-mediated actin cable assembly, based on ref. [4]. See main text for description of processes 1–6. (E) Schematic of the analytical model showing the actin and For3p populations, the allowed transitions, and rate constants. Cytoplasmic actin promotes dissociation of For3p from cell tips; For3p at cell tips promotes polymerization of actin monomers. (F) 2D slices from simulations of the 3D lattice model showing the actin monomer pool treated as a continuous field, cytoplasmic For3p dimers simulated as discrete subunits, and actin cables consisting of a continuum actin field and For3p speckles (superposition of 2 slices; image corresponds to PS1; cortical For3p not shown).

Many basic cell functions such as cell motility, endocytosis, cytokinesis, and establishment of cell polarity depend on the ability of actin proteins to polymerize into long filaments [1]. Actin filament nucleation and polymerization, followed by controlled disassembly, maintains actin subunits in a state of constant turnover between the monomer and filament states. This property provides cells with a highly dynamic and adaptable actin cytoskeleton that establishes patterns and forces within cells. Budding and fission yeast are model systems for the study of universal molecular mechanisms of actin polymerization [2]. The actin cytoskeleton of non-dividing yeast consists of two distinct components (see Fig. 1A): (i) “actin cables” which are bundles of actin filaments nucleated by formins that play a crucial role in establishing polarized cell growth by guiding the transport of secretory vesicles and organelles towards the cell tips [3], [4], [5], and (ii) “actin patches,” dense dendritic networks of actin filaments nucleated by the Arp2/3 complex that localize at sites of clathrin-mediated endocytosis [2], [6], [7].

Fission yeast formin For3p associates with transient cortical landmarks established by microtubules at cell tips where it nucleates actin filaments for cables [3]. Formins form dimers that remain processively attached at the growing barbed end of actin filaments and control their elongation rate by recruiting and transferring profilin-actin subunits to barbed ends [1]. Processive association of actin filaments to For3p presumably physically links the tip of the cable to protein complexes attached to the plasma membrane. Bundles of cross-linked filaments nucleated by For3p undergo retrograde flow away from cell tips towards the cell center [4], [5] where they disassemble through filament severing processes [2].

In live cells expressing For3p-3GFP from its native promoter, Martin and Chang [4] observed that the association of For3p with the cortical foci is transient: For3p dissociates from the tips of actin cables within seconds, forming dots which passively follow actin cable retrograde flow and disassembly in a turnover cycle similar to actin (Fig. 1A). On the basis of these observations, they proposed the mechanism shown in Fig. 1B–D. The movement of For3p away from the cortex was dependent on actin polymerization [4], indicating the existence of coupled control mechanisms between these two proteins. A similar behavior was reported for formin Bni1p that nucleates actin cables in budding yeast [8].

In this work we used recent estimates of actin and For3p concentrations (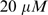 or 10^6^/cell for actin [9], and 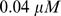 or 2·10^3^/cell for For3p [4]) to develop a quantitative model based on the processes in Fig. 1B–D. To our knowledge, this is the first modeling study of actin cable dynamics and of formin proteins in cells. We used the model to (i) test the validity and stability of the proposed mechanism, (ii) compare the model with experiment in quantitative terms, (iii) describe the model's dependence on the rate constants and protein concentrations, (iv) examine the implications of the coupled For3p and actin turnover in actin cable dynamics. Answering questions of global stability lead us to consider models at the whole cell level. We identified a combination of rate constants that reproduced the majority of relevant experimental observations, including morphological changes after treatment with Latrunculin A (LatA) and FRAP of For3p-3GFP. Our findings provide support for the mechanism of Fig. 1B–D, and generate predictions for the system behavior under changes in parameter values. We suggest experiments to help resolve some of the outstanding issues that our model helped reveal.

## Methods

### Model and Assumptions

We focus on interphase cells after “new end take off” (NETO) [10], when actin cables grow from both cell tips [11]. We assume that within the timescale of our interest (seconds to 2 minutes), actin cables grow out of stable cortical landmarks which are high molecular weight complexes involving Tea1p, Tea4p, and Bud6p [12]. Tea1p helps establish these foci through binding to growing microtubule ends that periodically touch the cell cortex near cell tips and deliver Tea1p locally [13]. Given that the rate of new microtubule end association with cell tips is ∼1/min [14], and ∼10 cables per cell tip [4], we estimate the lifetime of cortical foci to be several minutes, consistent with reported FRAP experiments of Tea1p [15].

For3p accumulates in large numbers at cortical foci in cells treated with LatA, suggesting that cortical landmarks provide multiple sites for For3p binding [3], [4]. For simplicity, we do not explicitly consider effects associated with saturation of cortical For3p binding sites. We assumed For3p forms stable dimers [16] throughout actin cables and the cytoplasm, though our results are not very sensitive to this assumption.

We developed two models at different levels of complexity: (i) a simple analytical model whose solution helps clarify the dependence of the system on parameter values, and (ii) a 3D computational lattice model that additionally accounts for the important effects of cytoplasmic actin and For3p diffusion, the effects of fluctuations in the small number of For3p molecules per cell, and allows direct comparison to prior experimental data. Both models consider explicitly the dynamics of For3p and actin only, collapsing the effects of regulatory [17], [18], [19] and other proteins into the values of rate constants.

#### (i) Analytical model

We assume that the total number of For3p dimers, 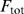, is distributed among three groups: 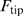, 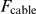, and 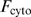, representing the total number of dimers at actin cable tips, along the actin cables, and diffusing in the cytoplasm, respectively. Similarly, the total number of actin subunits, 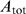, is distributed among filaments in actin cables, monomers in the cytoplasm, and filaments in actin patches, with numbers 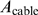, 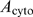, and 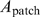, respectively (see Fig. 1E). In the analytical model we assume that 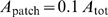 [9] is fixed.

Mass conservation and the following equation describe actin kinetics:
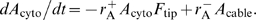
(1)Here, the first reaction term represents For3p-mediated actin polymerization at cable tips (see Fig. 1E) with rate 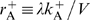, where 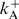 is the actin polymerization rate constant, 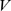 is the volume of the cell, and 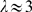 quantifies the enhancement of polymerization due to the excluded volume of organelles and macromolecular complexes in the cytoplasm [9]. We assume an effective linear dependence of polymerization rate on cytoplasmic actin monomer concentration, similarly to the approximately linear dependence of formin-mediated polymerization on actin monomer concentration at fixed profilin concentration [20], [21], [22]. The second reaction term in Eq. 1 describes actin cable disassembly at a constant rate 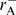. A more realistic, age-dependent depolymerization rate is used in the computational model below.

Denoting 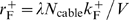, where 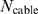 is the number of actin cables and 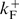 the effective rate constant for the binding of For3p dimers to a single cortical landmark, we describe the For3p kinetics as follows:
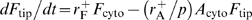
(2a)

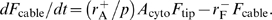
(2b)


The second reaction term in Eq. 2a and the first reaction term in Eq. 2b describe detachment of For3p from the cell cortex resulting in For3p becoming an inactive component of the body of the flowing actin cable. Martin and Chang [4] observed that after treatment with LatA, For3p accumulates in large numbers at cortical foci, it exhibits slower turnover as measured by FRAP, and moves with slower speed along actin cables; to capture these observations, we found that we had to assume that the rate of cortical For3p dissociation depends on the rate of actin polymerization (see last subsection of Results). The “processivity parameter” 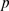 is the average number of actin monomers polymerized per cortical For3p dimer before detachment. The last term in Eq. 2b represents detachment of inactive For3p from cable bodies into the cytoplasm with rate 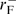.

#### (ii) Computational Model

We accounted for cytoplasmic diffusion using a 3D lattice model (cubic lattice, lattice site size 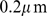, see Fig. 1F). We explicitly simulated the diffusion and reaction of individual For3p dimers on the lattice. Actin was modeled as a continuous field in the cytoplasm and actin cables. We modeled the cell as a tube of radius 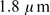 with two hemispherical caps at each end, a total length of 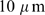, and volume 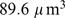 [9]. We assume that the actin cables grow out of 10 cortical sites at random positions on each of the hemispherical caps and that they are straight and parallel to the long axis of the cell. Actin cables buckle and bend, but we neglect these effects by assuming that they have little effect on actin and For3p turnover dynamics. In the absence of precise details on the ultrastructure of actin cables, we treat cables as a non-diffusive medium of non-uniform concentration, undergoing retrograde flow on a 1D lattice. We allow diffusion throughout the whole cell, accounting for the volume of organelles by multiplying rate constants by 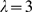 where appropriate. Since enhanced local concentrations are not equivalent to enhanced rate constants, crossovers between reaction and diffusion-controlled regimes are accurate to within prefactors of order 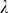 [23].

Processes 1–6 of Fig. 1B–D were modeled analogously to those of the analytical model, see Supporting Information Text S1. We do not account for the effect of myosin pulling, which may influence retrograde flow in budding yeast [5], [24], and assume that the flow rate is limited by actin polymerization. In addition, we considered two models for actin disassembly from cables: (i) uniform disassembly rate, 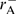, and (ii) an empirical Hill-type dependence of disassembly rate on the age 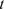 of a local actin cable segment: 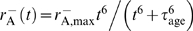, where 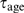 is a characteristic time for aging and 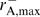 is the disassembly rate of fully aged filaments. Case (ii) accounts in a simple way for actin filament aging due to hydrolysis and phosphate release following ATP-actin polymerization, the preferable binding of cofilin to the sides of aged ADP-actin filaments, and for cooperative effects in network disassembly [25], [26]. We implemented similar mechanisms for For3p disassembly from cables. Cables were assumed to break at sites where their thickness is less than two actin filaments over 20 nm; broken segments were released as monomers in the cytoplasm.

A significant fraction of actin monomers is consumed in ∼50 [9] actin patches near the plasma membrane that assemble and disassemble within ∼25 s [6]. We modeled patches as stationary point sinks of constant strength during assembly over 12.5 s, and as point sources during disassembly. The strength of the sink is chosen such that each actin patch matures to ∼2700 actin subunits [9]. The patch is subsequently depolymerized linearly, releasing actin monomers into the cytoplasm. We initiate new patches near the plasma membrane at a constant rate and at random positions along the hemispherical cell tips (probability 60%) or randomly along the main body of the cell (probability 40%). In the model, actin patches contain ∼10% of the total actin on average.

## Results

### Comparison of Model to Experiment

We first used the models to (i) check the stability and self-consistency of the proposed mechanism, and (ii) compare the model to prior experiments in quantitative terms. We found that the analytical model has a single steady state. Denoting the fraction of For3p in the tips 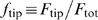, and the cytoplasmic actin fraction 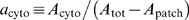, the solution for 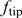 and 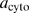 is a symmetric function of two dimensionless parameters, 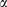 and 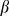:
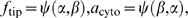
(3a)

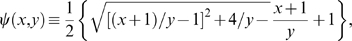
(3b)where
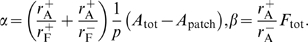
(4)The fraction of For3p in the cables is
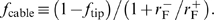
(5)Linear analysis indicates that the solution is stable, see Supporting Information Text S1.

**Figure 2:**
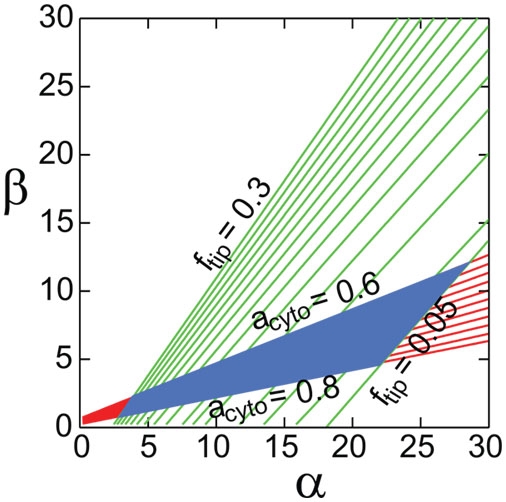
Results of analytical model. Fraction of For3p at cell tips, 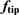 (green), and fraction of cytoplasmic actin, 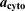 (red), as a function of 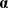 and 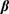 from Eqs. 3 and 4. The boxed region shows our estimate of the parameter range consistent with the physiologically realized case: 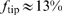 (10 For3p dimers/cable, 20 cables/cell and 1500 For3p dimers/cell) and 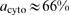 (assuming cables of 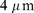 in length, cable thickess of 10 actin filaments, and 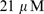 total actin concentration with 10% in patches), see Table 1.

Estimating 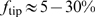 and 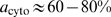, in Fig. 2 we outline the allowed region in parameter space corresponding to steady states consistent with this range (α∼5–23 and β∼1–7). In agreement with this requirement, an estimate of the values of rate constants gives 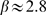 (see Table 1). Since 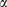 depends on less certain parameters (such as 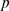, 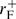 and 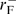), the system may lie in the allowed region of Fig. 2A with multiple combinations of rate constants. Thus the analytical model captures the general features of the actin and For3p partitioning among components but additional constraints are required to to pin down possible values of rate constants. We used 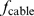 in Eq. 5 and the results of the more complex computational model that depend on parameter values in a more involved manner than a simple function of 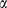 and 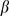 to determine the range of allowed parameters. We thus identified a combination of rate constants that is the most consistent with the set of available experiments (“Parameter Set 1”, PS1), see Table 1. The quantitative agreement of PS1 to experiment provides support for the model. Two less successful sets of parameters, Parameter Sets 2 and 3 (PS2, PS3), are discussed in a separate subsection below.

**Table 1:** Model parameters (Possibility 1).

Symbol	Description	Value	Symbol	Description	Value
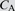	Global actin concentration	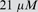 [9]	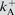	Actin polymerization	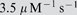 d
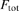	Total number of For3p dimers	1500^a^	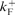	For3p cortical association	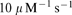 e
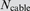	Total number of actin cables	20^b^	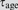	Actin filament aging time	5 s f
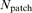	Average number of actin patches	50^b^	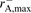	Aged actin filament disassembly rate	0.4 s^−1^ f
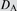	Actin monomer diffusion coefficient	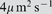 [40]	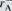	Uniform actin disassembly rate	0.1 s^−1^ g
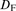	For3p dimer diffusion coefficient	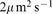 c	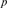	For3p processivity	2000^h^

a Using a slightly larger number than 1800 For3p/cell [4] to obtain realistic numbers of For3p dimers per cable tip (see Fig. 3A).

b From published images [4], [27], [9].

c Estimate, using a value smaller than 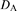 to account for the larger size of For3p compared to actin monomers.

d Value reproducing measured cable flow rates.

e A fraction 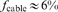 is required for the density of For3p dots along actin cables in the simulations to be consistent with experiment (Fig. 1A). Using 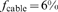, our estimate 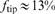 (see Fig. 2A), and 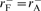 in Eq. 5, we estimate 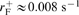 which corresponds to 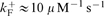.

f Value reproducing actin cable lengths and density profiles along actin cables that are consistent with experiment [8], [4]. For3p disassembly rates are identical to those of actin.

g Value for which the analytical model and the computational model with age-independent disassembly give identical results in the limit of fast cytoplasmic diffusion coefficients.

h Value required to obtain a density of For3p dots along actin cables consistent with experiment, corresponding to 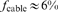. Consistently with this, using the values of the table in Eq. 4, the bounds of Fig. 2A require 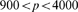.

#### Parameter Set 1: Large processivity parameter, slow For3p association to cable tips, and slow dissociation from cables

Assumptions of PS1: (i) For3p disassembles from actin cables with the same rate as actin subunits, and (ii) the fraction of For3p along cables has a value 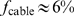 that reproduces For3p dots as in Fig. 1A. Our estimate of 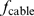 is obtained from the images of ref. [4] that indicate 3–5 For3p dots per cable and thus a total of 60–100 For3p dimers along the cables (for 20 cables/cell). The parameter values corresponding to PS1 are listed in Table 1. We find that 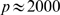, i.e. each cortical For3p dimer polymerizes thousands of actin subunits before detachment into the cable.

Figure 3A,B shows that the average number of For3p dimers per cable tip (corresponding to the number of actin filaments in the cable), and cable flow rate fall into the observed range for a wide range of values of the number of For3p dimers per cell. Experimentally, the number of For3p dimers per cable tip in wild type cells ranges from ∼5 to 20 [4], [27] while the retrograde flow rate ranges from 0.1 to 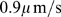 with an average of 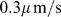 [4], [5], all consistent with Fig. 3A,B. In the figure, the number of For3p at cable tips increases with increasing concentration of For3p due to (i) higher rates of For3p association with the cortex, and (ii) depletion of the actin monomer pool by For3p which results in smaller rates of cortical For3p dissociation. The latter effect is stronger for smaller diffusion coefficients of actin due to additional depletion of actin near cell tips (see below). Actin monomer depletion also causes the retrograde flow rate to decrease as the total number of For3p is increased (Fig. 3B). The computational model essentially gives the same results as the analytical model in the limit of large cytoplasmic diffusion coefficients (Fig. 3A,B). In this limit, cytoplasmic concentrations become essentially uniform, as assumed in the analytical model.

**Figure 3:**
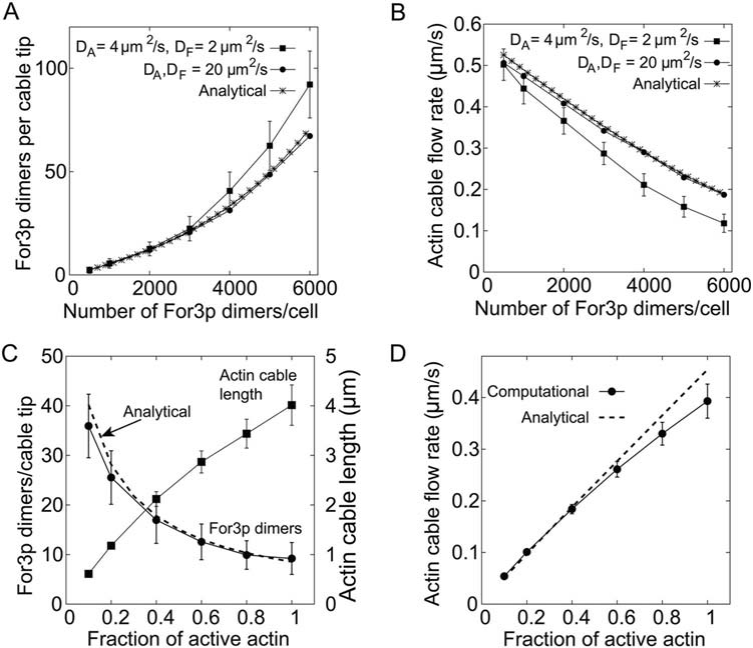
Results of the computational and analytical models using the parameters of Table 1. (A) Average number of cortical For3p dimers per cable, as a function of For3p concentration. In the limit of large cytoplasmic monomer diffusion coefficients, the results are close to those of the analytical model. (B) Cable flow rate as a function of For3p concentration. For large diffusion coefficients, we find agreement with the results of the analytical model. (C) Average number of cortical For3p dimers per cable and actin cable length as a function of the fraction of active cytoplasmic actin. Decreasing fractions simulate the effect of increasing doses of LatA. The dashed line shows the corresponding calculation using the analytical model. (D) Actin cable flow rate vs. fraction of active actin. The error bars in A–D show the standard deviation among all actin cables over 100 s.

To compare with the results of experiments of cells treated with LatA [3], [4], we simulated the effects of LatA as a reduction of the fraction of active cytoplasmic actin, which is equivalent to a reduction of the actin polymerization rate constant, 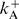. Fig. 3C shows that increasing doses of LatA (lower fraction of active actin monomer pool) result in accumulation of For3p at cable tips because the rate of detachment of For3p from the cell cortex decreases with decreasing actin polymerization rate. Lower fractions of active actin also cause shortening of cables (Fig. 3C), and slowing down of cable retrograde flow (Fig. 3D). The results of Fig. 3C,D are in agreement with the observations in ref. [4].

The model predicts noticeable fluctuations in the number of For3p dimers at cable tips (Fig. 3A,C). These fluctuations are not large enough to fully destabilize the cable by fluctuating down to zero. The retrograde flow rate of actin cables also exhibits fluctuations (see Fig. 3B,D), reflecting the spatial and temporal fluctuations in the cytoplasmic actin pool which is non-uniform and changes in time. Cytoplasmic actin concentration is lower near actin monomer sinks such as regions at cell tips locally rich in cortical For3p. The strength of the sink at each actin cable tip is fluctuating, providing an additional contribution to fluctuations in flow rates. Actin patches, which act as random sinks or sources for actin in the cytoplasm, also contribute to fluctuations of flow rates by disturbing local actin concentrations. The magnitude of our observed fluctuations in flow rate is comparable but somewhat smaller to the experimentally measured spread of 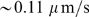 [4]. This is consistent with our model, however, since these experimental measurements involved multiple cells whose actin and For3p concentrations were different.

**Figure 4 pone-0004078-g004:**
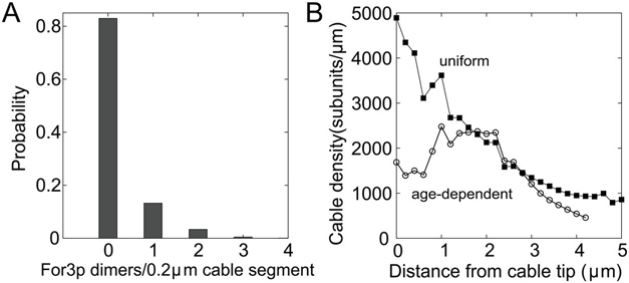
Densities of For3p and actin along actin cables. (A) Probability distribution of the number of For3p dimers per actin cable segment; actin cables were divided into 1D segments of order the diffraction limit (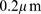) as in Fig. 1F. The corresponding fraction of For3p in cables is 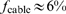. (D) Typical actin cable density profile assuming uniform (▪) or age-dependent () depolymerization rates.

The model successfully generates For3p dots which move along the cable and occur with a frequency similar to experiments with the chosen 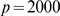 (see Fig. 1A,F). A For3p dot was found to contain on average 2.1±0.8 For3p dimers (ranging 1–10) [4], likely due to the limited sensitivity in detecting single dimers. We find that the number of two or more For3p dimers within a distance smaller than the diffraction limit is ∼0.8 per cable for a typical cable of length 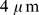 (see Fig. 4A). Given the uncertainties involved, this number is within the range allowed by experiment [4].

In studies of budding yeast, Buttery et al. [8] observed that the intensity of actin cables labeled with phalloidin fluctuates strongly around an average value along the cable. The computational model reproduces a similar pattern for the actin density along the cable (see Fig. 4B). This fluctuating pattern is due to two combined effects: (i) the fluctuating number of For3p dimers at cable tips (Fig. 3A) leads to polymerization of actin cables of non-uniform intensity, and (ii) our assumption of an age-dependent cable depolymerization mechanism. Since newly polymerized actin is protected from disassembly, the For3p-induced changes in thickness at the cable tips can propagate in an undistorted manner along the cable. For comparison, the actin density of cables generated by age-independent disassembly decays exponentially away from the cell tip in a manner which appears inconsistent with ref. [8] (Fig. 4B). These results show that the kinetics of aging of polymerized actin subunits may play an essential role in maintaining stable actin cables [25], [28].

**Figure 5:**
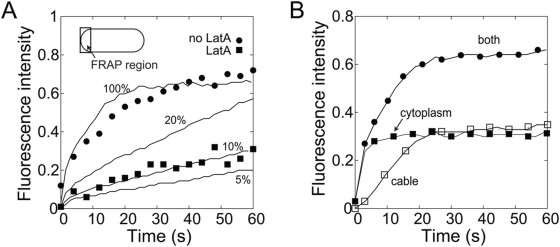
Comparison of simulated FRAP curves to experiment, using parameters from Table 1. Curves are normalized to unity before simulated bleaching of a region near cell tips at 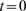. (A) FRAP curves as a function of decreasing active cytoplasmic actin concentration to simulate the effects of increasing doses of LatA. Each curve is the average of 30 runs. The results are consistent with the data reproduced from ref. [4]. (B) Plot of the FRAP curve of panel A (100% active actin) showing the separate contributions of For3p in the cytoplasm and in the cables.

We used the model to fit and interpret FRAP experiments of For3p-3GFP in a region of size 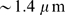 near cell tips [4]. We simulated these experiments by marking all For3p dimers inside the hemispherical cap at one tip of the cell as photobleached at 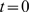, and recording the number of unmarked For3p in the same region over time. The simulated FRAP curves agree very well with the experiment in both normal cells and cells with sequestered cytoplasmic actin (simulating LatA treatment), see Fig. 5A. Fig. 5B shows the relative contributions of For3p in the cytoplasm and in actin cables (at both tips and cable body) to FRAP. The total recovery is almost equally split into the two contributions. Cytoplasmic recovery dominates at short times (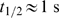), while recovery of For3p at cables is slower (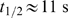) and exhibits a lag phase. The recovery of For3p in the cytoplasm is mainly due to diffusion while cable recovery depends on the slower rate of detachment of For3p from the cell cortex. Thus, we interpret the recovery time of 10 s measured in experiments [4] as the combined effect of both diffusion and For3p detachment. In our simulations, two factors cause the slow recovery of For3p in cells treated with LatA: (i) the rate of detachment of cortical For3p becomes smaller due to the decrease in the polymerization rate, and (ii) the fraction of cortical For3p increases relative to the cytoplasmic For3p which recovers at the same fast rate as in cells without LatA. The simulations are also consistent with the reduction in the magnitude of the total percent recovery in cells treated with LatA, due to the photobleaching of a larger fraction of the total For3p in the cell. The best fit for cells in LatA is obtained for 10% active actin. LatA has been estimated to bind to both actin and profilin-actin monomers with a dissociation constant in the range 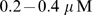 in vitro [29], [30]. Such a value would imply that only 0.03% of actin monomers remain free at 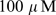 LatA, the concentration used in ref. [4]. However residual actin polymerization may have persisted in these cells, since a For3p mutant (I930A) that cannot bind to actin barbed ends had undetectable recovery in LatA [4].

### Predictions of System Behavior

Having tested the validity of the mechanism of Fig. 1A as a quantitative description of actin cable dynamics, we now use the model to describe the response of the system to changes in parameter values in order to (i) suggest experiments for further tests of the model, and (ii) provide insights on the biological mechanisms of actin cable control. Focusing on PS1, in the following we display results for the three cable parameters which are likely to be the most significant for the cell: number of cortical For3p per cable (related to cable thickness), actin cable length, and actin cable flow rate. The analytical expressions of Eqs. 3–5 are an additional guide for the partitioning of actin and For3p among actin cables, cable tips, and cytoplasm and their dependence on parameter values. To enable the readers to visualize the results of changes in parameter values beyond those in the main text and Supporting Information, a graphical Java applet simulation of the model is available at http://athena.physics.lehigh.edu/research/actin_cable_applet.html.

**Figure 6:**
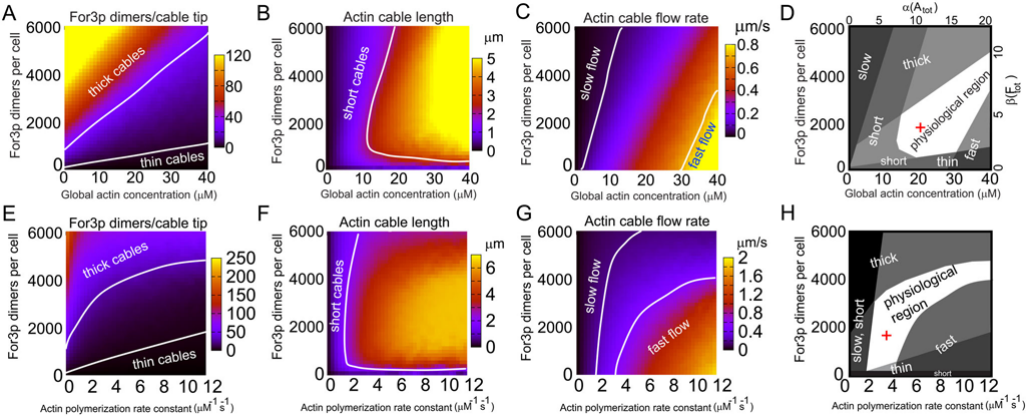
Plots of observables obtained from the computational model using the numbers in Table 1. (A)–(C) Average number of cortical For3p dimers per cable, cable length, and retrograde cable flow rate as a function of actin and For3p concentrations, with all other parameters (such as cell volume) fixed. The plots identify regions in which model results fall outside the range allowed by experimental observations. Cable length is a non-monotonic function of For3p concentration since: (i) large For3p concentrations deplete the actin monomer pool, slow down actin polymerization, and thus reduce the length of filament since we assumed that the severing mechanism is unchanged, and (ii) low For3p concentrations result in thin actin cables which are more likely to break due to our assumption of breaking of thin cables. (D) Plot showing the superposition of the excluded regions of plots (A)–(C) in gray. Expressed in terms of parameters 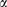 and 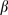, the allowed white region is similar to the expectations of the analytical model, Fig. 2A. The red cross shows the point corresponding to the values of Table 1. (E)–(H) Same as panels A–D, showing the dependence on actin polymerization rate constant, 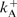, and For3p concentration. Panel H is qualitatively similar to panel D, but the dependence of the observables on 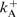 is weaker for large concentrations of For3p due to the onset of cytoplasmic actin concentration gradients (see main text).

Fig. 6A–C shows the dependence of the number of For3p dimers per cable tip, cable length, and cable flow rate on the total concentrations of actin and For3p, with all the other parameters having the values shown in Table 1. In the figure we identify regions in which the actin cables become unusually short, thick or thin, or undergo very fast or slow retrograde flow. Fig. 6D indicates the region of parameter space in which the values of the parameters plotted in Fig. 6A–C fall within the experimentally observed range. In terms of 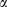 and 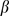, the allowed region is consistent with the allowed regions in Fig. 2 and Fig. S1 that are based on a comparison of the predicted actin and For3p partitioning among components to experiment. The model predicts an optimal concentration of For3p for maximal cable length (Fig. 6B): high levels of For3p deplete the actin monomer pool which results in slow cables that age and depolymerize when they are still short, while low For3p levels generate very thin cables that have a high fragmentation rate.

Fig. 6E–H shows the model's results as a function of the actin polymerization rate constant, 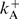, and the concentration of For3p. At small For3p concentrations, the effect of an increase in 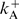 is qualitatively similar to the effect of an increase in the total actin concentration shown in Fig. 6A–D. In the limit of high For3p concentrations, however, the behavior in Fig. 6E–H is more weakly dependent on 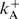 than the dependence on actin concentration in Fig. 6A–D. As a result, the regions of Fig. 6H are distorted versions of those in Fig. 6D. Hence the effect of LatA, which can be approximated as a reduction of 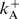, is not identical to a decrease in the total actin concentration. The origin of the differences between Fig. 6A–D and Fig. 6E–H is the development of concentration gradients in the cytoplasm with increasing For3p concentration (see below).

**Figure 7:**
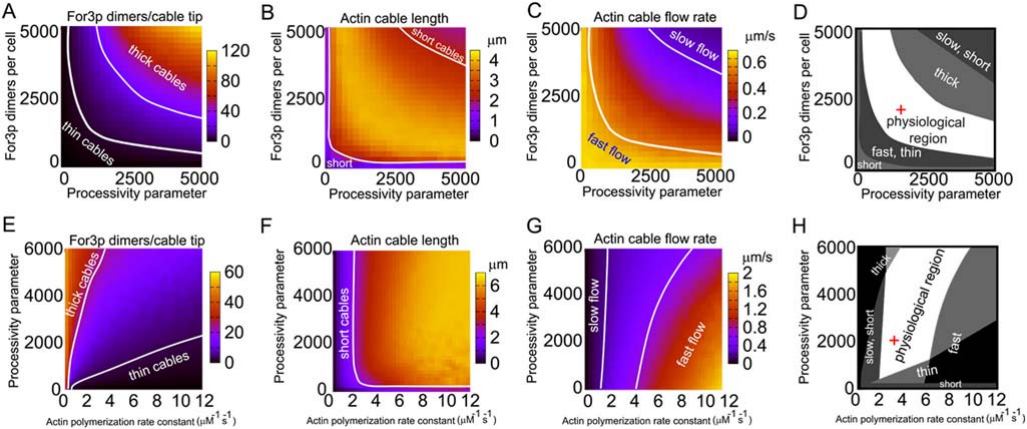
Same as Fig. 6, showing the dependence of average number of cortical For3p dimers per cable, cable length, and retrograde cable flow rate on processivity parameter 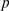 and For3p concentration (panels A–D), and on processivity parameter and 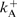 (E–H). The non-varied parameters are those in Table 1.

An important parameter of the model is the processivity parameter 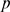. Fig. 7 shows the predicted dependence of the results of the model on 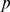 and For3p concentration (Fig. 7A–D), and on 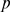 and actin polymerization rate constant (Fig. 7E–H). The behavior of the observables in Fig. 7A–D indicates that an increase (decrease) in For3p concentration can be balanced by a corresponding decrease (increase) in the value of the processivity parameter. Thus processivity and For3p concentration play a similar role. This explains why the behavior shown in Fig. 7E–H is similar to the structures in Fig. 6E–H.

**Figure 8:**
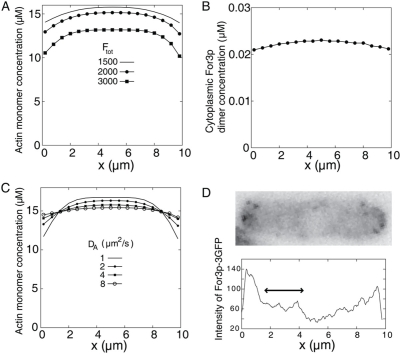
Actin and For3p concentration profiles along the long axis of the cell. (A) Simulated time-averaged concentration profile of actin monomers in the cytoplasm, using parameters from Table 1. A noticeable concentration gradient develops near cell tips. Increasing concentration of For3p depletes the actin monomer pool, and steepens the profile over a narrower region near the tips (since the cables become shorter). (B) The cytoplasmic For3p profile is less steep than that of actin. (C) The actin monomer profile depends on the values of the actin monomer diffusion coefficient, 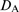, becoming steeper as 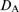 becomes smaller. (D) Top: Image of a cell expressing For3p-3GFP, single frame of Movie 2 of ref. [4] (reproduced with permission). Bottom: Average intensity profile along a strip of width 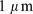 across the long axis of the cell above. We obtained the values in the graph by inverting the image and subtracting the background intensity outside of the cell.

Our model predicts that the retrograde flow of actin cables is strong enough to induce significant concentration gradients of cytoplasmic actin monomers along the long axis of the cell (Fig. 8A). The origin of the gradient is easily seen by considering the balance between the actin flux due to retrograde flow towards the cell center with the diffusion of actin monomers in the opposite direction. The retrograde flux is approximately equal to the total rate of actin polymerization at one of the cell tips: 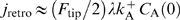, where 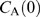 is the actin monomer concentration at the tip. The diffusive flux across a cross section of the cell is 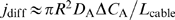, where 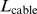 is the average actin cable length, 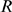 is cell radius, and 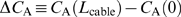 is the difference in actin monomer concentration between the cell tip and a position at a distance 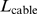 away (assuming a linear gradient and that the cables growing from either tip do not overlap at the center of the cell). Using a similar argument for the cytoplasmic concentration of For3p, 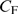, one has
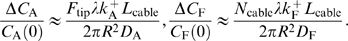
(6)Using the parameters of Table 1 and 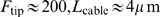 (Fig. 3C), we find that the cytoplasmic actin (cytoplasmic For3p) concentration is 13% (10%) higher at the cell center as compared to the cell tips, close to the numerical results in Fig. 8 (17% and 10%, respectively). The gradient in actin monomer concentration is steeper than that of For3p (see Fig. 8B) since the corresponding reaction sink term in Eq. 6, 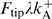, is larger than 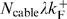, assuming similar diffusion coefficients for actin and For3p.

Fig. 8A shows that the gradient in actin monomer concentration becomes steeper with increasing total concentration of For3p. Thus, diffusive flux towards the cell tips becomes the limiting factor for polymerization at sufficiently high concentrations of For3p. This transition from reaction to diffusion-controlled kinetics is reflected in the weak dependence of the results of Fig. 6E–H on the rate constant 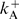 in the limit of high For3p concentrations. As expected from Eq. (6), the actin monomer concentration gradient becomes steeper as the diffusion coefficient of actin becomes smaller, and becomes flat as the diffusion coefficient becomes large (see Fig. 8C). The actin monomer concentration gradient is stable: over 2 minutes, our numerical simulations indicate that the slope of the gradient has a relative error of magnitude 20%. To our knowledge, there have been no measurements of the cytoplasmic actin monomer concentration profile in these cells.

Fig. 8D shows the intensity profile of For3p-3GFP in a typical cell from ref. [4]. This image represents the total distribution of For3p in the cell, including cortical and cable For3p. Cortical For3p is likely the main origin of the intensity peaks at the tip regions. The region marked with a double arrow represents the part of the cell whose intensity is expected to be dominated by cytoplasmic For3p-3GFP. This region does not exhibit a noticeable gradient, consistently with Fig. 8B (but not with PS3, see next section). Since we do not explicitly account for the exclusion of For3p from the nucleus, the model does not generate a depletion of For3p at the center of the cell as in the image of Fig. 8D.

### Other Parameter Sets

**Figure 9:**
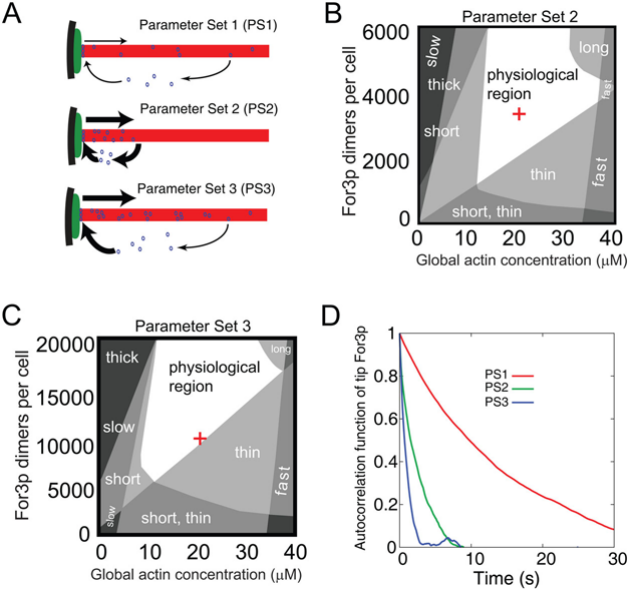
Results for Parameter Sets 2 and 3. (A) Schematic of PS1, PS2, PS3 (Tables 1, S1, and S2, respectively). The turnover of For3p at cable tips in PS2 and PS3 is much faster than in PS1. The rate of For3p dissociation from cables in PS2 is much faster than in PS1 and PS3. Formation of For3p dots as in Fig. 1A is typical for PS1. Long-lived dots do not form in PS2 and in this case the observation of dots in experiments would be attributed to unlikely events, not included the model. In PS3, a highly fluctuating concentration of For3p in the cables establishes a speckled pattern along the cable. (B) Dependence of cable thickness, cable length, and retrograde cable flow rate on the total concentrations of For3p and actin for PS2, as in Fig. 6D for PS1. (C) Same as panel B for PS3. (D) Plot of the autocorrelation function of cortical For3p as function of time for PS1, PS2, PS3. Since the cortical For3p dissociation rate is smaller in PS1 as compared to PS2 and PS3, the PS1 autocorrelation function decays more slowly as compared to PS2 and PS3 with a half life of several seconds. Experimental measurements of the cortical For3p autocorrelation function may help distinguishing among PS1, PS2, and PS3.

In PS1, each For3p dimer polymerizes ∼2000 subunits before cortical detachment. Thus additional mechanisms are required to explain the short length of actin filaments observed in electron microscopy images of actin cables, which consist of filaments of ∼100 subunits each [27]. Filament severing by proteins such as cofilin or filament fragmentation during sample preparation may generate short filaments out of longer ones. We find that a mechanism in which the short length of filaments is primarily due to For3p detachment cannot be rigorously excluded, though such a mechanism is less consistent with the full set of available experimental data. We found two parameter sets, PS2 and PS3, in which the filament length in the cables is limited by the detachment of cortical For3p, i.e. 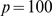 (see Fig. 9A).

#### Parameter Set 2: Small processivity parameter, fast For3p association to cable tips, fast For3p dissociation from cables, and slow cytoplasmic diffusion of For3p

The rate constants of PS2 are listed in Table S1 whose main features are as follows. (i) For3p dissociates from actin cables faster than actin subunits, with a rate 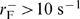 such that the body of the actin cable is not saturated with For3p. (ii) For3p binds to cortical landmarks with a rate constant 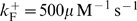 to maintain the required population of For3p at cable tips. Such a high 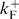 value could be reached by a large number of cortical binding sites for For3p. (iii) The cytoplasmic diffusion coefficient of For3p is much smaller than that of actin, 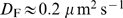, to fit the FRAP data. In Supporting Information (Fig. S3) we show that PS2 can be used to interpret most of the experimental results described in Figs. 3–5 for PS1. However, the main problem of PS2 is that it does not generate For3p dots moving along actin cables as in Fig. 1A (see Fig. S3H). In PS2 the appearance of For3p dots would need to be attributed to unlikely events involving long-lived For3p on actin cables. In Fig. 9B we show the dependence of the physiological properties of actin cables on the total concentrations of actin and For3p, in analogy to Fig. 6D. The structure of the the regions of Fig. 9B is similar to that of Fig. 6D, though the precise shapes are modified.

#### Parameter Set 3: Small processivity parameter, fast For3p association to cable tips, slow For3p dissociation from cables, and slow cytoplasmic diffusion of For3p

Assumptions: (i) 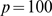 for for the same reasons as in PS2, and (ii) For3p disassembles from actin cables into the cytoplasm with the same rate as actin subunits. The full set of PS3 rate constants is shown in Table S2, whose main difference as compared to PS2, other than low For3p dissociation rate from cables, is higher total concentrations of For3p (10^4^ dimers/cell). In Fig. S4 we show how PS3 can be used to interpret the available experimental results. In PS3, the body of actin cables has a dense but very spotty stream of For3p (see Fig. S4H). The origin of the large fluctuations in For3p density along the cables is the combined stochasticity in both the association and dissociation of For3p at cable tips. This pattern could conceivably be consistent with the observations of Fig. 1A if the experimental detection sensitivity was at the level of ∼6 For3p dimers instead of 2 as assumed in PS1. However, the expected occasional appearance of small For3p streams is not evident in Fig. 1A, making the validity of PS3 less likely. Unlike PS1 and PS2, in PS3 the large rate of For3p retrograde flow generates an extended gradient in the cytoplasmic concentration of For3p (see Fig. S4F). This gradient appears however to be inconsistent with the image of Fig. 8D. Fig. 9C shows the dependence of the physiological properties of actin cables on the total concentrations of actin and For3p.

### Importance of For3p Detachment Mechanism

A main component of the model is the detachment step of For3p from the cell's cortex. In the model, the rate of cortical For3p dissociation increases linearly with the rate of actin polymerization. Such an increase is required for the results to be consistent with most prior observations. We found that a model in which the rate of cortical For3p dissociation is independent of the rate of actin polymerization cannot explain the increase in cortical For3p intensity after LatA treatment [4], unless the system's parameters are close to those of PS3 (with the exception of parameter 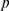 which has no meaning in this case), see Fig. S5. In PS3, a cytoplasmic For3p gradient is maintained at steady state by the rapid transport of For3p away from cell tips by actin cables (see Fig. S4H). This gradient disappears in the presence of LatA which depolymerizes the cables, thus allowing more of For3p to associate with the cortical foci at the tips. As described above, however, PS3 is the most problematic parameter set since it also requires a small diffusion coefficient for For3p, a re-examination of the For3p concentration measurements of ref. [4], and the existence of a concentration gradient in cytoplasmic For3p which is not evident in images.

The value of 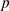 is in the range of 100–5000, depending on PS1, PS2, and PS3. These values are consistent with in vitro experiments in which the processivity parameters for various formins (but not for For3p whose polymerization properties have not been studied in vitro) were found to be a few times larger, ∼2·10^4^ [20], [22]. Additional factors such as internal stresses at the tips of actin cables may contribute to dissociation in cells. An interesting possibility (that could simultaneously explain the inactivity of For3p within cables) is that cortical dissociation occurs when For3p becomes trapped within the body of a growing filament as a result of unsuccessful stepping of its FH2 domain, as suggested [22].

More complex dependencies of the rate of cortical For3p detachment on polymerization can lead to additional phenomena, such as the existence of multiple steady state concentration profiles in the first of Eq. 6. To see this, let us assume that the processivity parameter 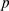 depends on the local concentration of actin monomers, 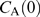. For simplicity, let us also assume that the cytoplasmic concentration of For3p is essentially uniform, as in PS1 (Fig. 8B). At steady state, from Eq. (2a) applied at the tip position, 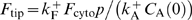. Assuming a uniform actin cable disassembly rate, 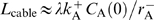, where 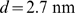 is the increase in filament length per actin monomer polymerization event. Substituting in Eq. 6, one has
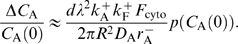
(7)Assuming a given cytoplasmic concentration of actin monomers and For3p at the center of the cell, this equation can have two solutions for 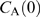, provided that 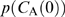 is complex enough. This would imply the existence of solutions such as monopolar distribution of actin cables and cortical For3p. In this case, each tip of the cell could share the same cytoplasmic concentrations of For3p and actin at the center, but can have different actin concentration and cortical For3p at each tip, even when the distribution of cortical For3p binding sites is symmetric at both tips. This appears to be the situation during monopolar growth, before NETO [11]. This effect would be due to a bistability in the antagonistic role of For3p which removes actin monomers from the cytoplasm, and actin monomers which promote removal of For3p from the cortex. In future work, we plan to explore the plausibility of these effects and their possible relationship to monopolar growth and NETO, and to compare to other models [31]. Similar effects may arise if parameter 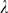 in eq. 7 has a strong dependence on local actin cable density.

## Discussion

Since actin cables are structures whose dynamics can be monitored by fluorescence microscopy, and since yeast is a tractable genetic system, comparison of the results of theoretical models of actin cables to experiment could be one of the best choices to help us understand the power or the limits of predictive theoretical modeling of the cell cytoskeleton of live cells. Our work is a first model of actin cable polymerization that generates quantitative predictions on the functional relationships among the components of the actin cable system, based on detailed comparison to prior experiments. The predictions of the response of the actin cable system to variations in the concentration of For3p (Figs. 3, 6 and 7) could be tested in future experiments involving For3p overexpression and/or systematic reduction of For3p expression levels. The results of Fig. 6 and 7 showing the effects of changes of the polymerization rate constant and processivity parameter could be tested by targeted changes in the FH2 and FH1 domains of For3p that mediate polymerization and processive motion [20], [21], [22], [32]. The above could be combined with treatments with drugs such as LatA which results in an effective reduction in the value of the actin polymerization rate constant.

The three scenarios PS1, PS2, PS3 provide a very different kinetic picture of For3p function. These possibilities bring together a number of ideas that have been proposed in the field of actin cables and show three ways in which they can be combined to interpret prior experimental observations. While PS1 appears to be a much more successful parameter set, PS2 and PS3 cannot be strictly excluded. Comparison of the numbers in Tables 1, S1, and S2 and Fig. S2, S3, S4 shows that measurements of cytoplasmic diffusion coefficients of For3p (e.g., by fluorescence correlation spectroscopy or cytoplasmic FRAP), tests of cytoplasmic concentration gradients, and improved protein concentration measurements will help to further distinguish among the three cases. Measurements of the autocorrelation function of the number of For3p dimers at cable tips (e.g. by imaging the intensity changes of cortical For3p-GFP) may also also help distinguish among the three possibilities (see Fig. 9D).

Despite its low concentration, we estimate that For3p nucleates approximately the same amount of filamentous actin as the much more abundant Arp2/3 complex in actin patches. In addition, by generating long range transport of actin across the cell, For3p can induce concentration gradients of actin monomers and For3p in the cytoplasm. Cytoplasmic gradients may be essential in establishing dynamical landmarks to maintain cell polarity [33] and our model suggests that actin and For3p may be directly involved in these mechanisms. Even though the gradient in actin monomer concentration is small, its possible coupling to other regulators (such as regulators of microtubule polymerization dynamics) may help amplify the tendency of the system to self-polarize. In this work we assumed the system was already polarized by placing cortical foci at the tips of the cell.

Cells may have optimized the actin cable parameter values to achieve robustness [34]. This presumably corresponds to maximum size of the physiological region in Fig. 6, 7 and 9. However, the actin cable system is also highly adaptable, since the actin cytoskeleton undergoes large changes during the cell cycle (e.g. during cytokinesis actin filaments move to the cell's center as opposed to the ends [35]). It is possible that the size of the physiological region is large enough to allow robust behavior, yet small enough to allow for changes. For example, the reason why cells may have chosen PS1 as opposed to PS2 or PS3 may be that the actin cable morphology in Fig. 6D is more sensitive to an increase in For3p concentration as compared to Fig. 9B and 9C. Our study motivates systematic experimental exploration of parameter space to test these issues. Such studies will also help reveal more quantitative details on the precise role of other components of actin cables which were not explicitly included in our work, such as regulatory pathways and bundling kinetics.

The system closest to fission yeast is budding yeast in which formins Bni1p and Bnr1p nucleate actin cables. During bud growth, Bni1p localizes at the tip of the bud while Bnr1p localizes at the bud neck [2]. The rate of cortical detachment is fast for Bni1p and very slow for Bnr1p [24]. The rate of Bni1p detachment appears comparable to the rate of detachment of For3p; thus Bni1p may operate similarly to For3p. Overexpression of full length Bni1p or unregulated forms of Bni1p leads to reorganization of the actin cytoskeleton in a manner which is consistent with the results of Fig. 6A, B: upon Bni1p overexpression the actin cables become shorter and more dense within the bud [36], [37]. In these overexpression studies, the actin cables within the mother cell (presumably nucleated by Bnr1p) become short and thin [36], though some mother cells become unusually large and contain multiple cable-like fragments [37]. This change in the actin cables in the mother cell could be due to the Bni1p-induced depletion of the actin monomer pool available to Bnr1p. The observed trend, short and thin cables in the mother, is different to the prediction of short and thick actin cables in Fig. 6A,B upon reduction of the actin monomer concentration. This difference, however, is consistent with the difference between the detachment rates of For3p and Bnr1p [24]. Unlike For3p, the observation of thin cables in the mother may indicate a slight increase in the rate of Bnr1p cortical detachment with decreasing actin polymerization rate. Because of uncertainties in the mechanisms of Bnr1p cortical dissociation and association, the effects of Bnr1p overexpression [38] are harder to interpret with our model. Full length Bnr1p overexpression has small effects [38], though overexpression of unregulated Bnr1p leads to serious defects that can be rescued by an increase in the concentrations of proteins that bind to actin monomers or with treatment with LatA, possibly by reducing Bnr1p-mediated nucleation of actin filaments in the cytoplasm [38].

Our results may have implications on the general role of formins in cells beyond fission yeast. Since changing parameter values establish different distributions of actin and For3p within yeast, many other eukaryotic cells may have also used this property to establish different patterns and structures. Future work will uncover the extent of universality in the mechanisms of formin function. Much remains to be established, for example, on the precise function of fission yeast formin Cdc12p in nucleating disperse actin meshworks and/or actin cables during the assembly of the cytokinetic contractile ring [35], [39]. Hopefully, the modular structure of biological systems will allow us to proceed to a hierarchical understanding of the cell biological function of formins, starting from general features at a mesoscopic level of description as in this work, down to the full details of regulatory pathways that may differ across organisms.

## Supporting Information

Text S1 (0.03 MB PDF)

Table S1 (0.02 MB PDF)

Table S2 (0.01 MB PDF)

Figure S1 Fraction of For3p at cell tips, *f*_tip_, (green) and fraction of cytoplasmic actin, *a*_cyto_, (red) as a function of parameters *α* and *β* using the computational model. The total concentrations of actin and For3p dimers are varied, with other parameters fixed as in Table 1 (Parameter Set 1). The regions colored red and green show the regions in which 0.6<*a*_cyto_<0.8 and 0.05<*f*_tip_<0.3, respectively, with the overlapping region in orange. The physiological region is similar to the analytical model (see Fig. 2A in the main text).
(0.27 MB PDF)

Figure S2 Summary of simulation results of Parameter Set 1 (Table 1). (A)–(B) Dependence of the number of For3p dimers at cable tips, actin cable length, and actin cable flow rate on the fraction of polymerizable actin monomers. The reduction in the fraction of active actin monomers was simulated as a reduction of the polymerization rate constant *k* ^+^_A_ to mimic the effects of LatA. The trend is consistent with the experiments of Martin and Chang. The error bars show the standard deviation among all actin cables over 100 s. (C)–(D) Simulated FRAP curves of For3p at cell tip, same as Fig. 5. (E)–(F) Cytoplasmic concentration of actin and For3p along the length of the cell, same as Fig. 8. (G) Example of actin filament density along an actin cable, same as the age-dependent curve of Fig. 4B. (H) A snapshot of a 2D slice from the simulation showing the continuum actin field and For3p dots along actin cables.
(0.19 MB PDF)

Figure S3 Summary of simulation results of Parameter Set 2 (Table S2), same plots as in Fig. S2. (A)–(B) PS2 exhibits similar trends to PS1 for the number of For3p dimers per cable tip, average cable length, and average cable retrograde flow rate. (C)–(D) The simulated FRAP curves of For3p near the cell tip fit the experimental data in the presence and absence of LatA simulated as a reduction of active cytoplasmic actin (numbers next to curves). Compared to PS1, the recovery of For3p at cell tips is dominated by cytoplasmic For3p due to the relatively low fraction of For3p at cable tips and cable body. (E)–(F) Similarly to PS1, the cytoplasmic concentration of actin exhibits a concentration gradient, and the cytoplasmic concentration of For3p is approximately uniform along the cell. (G) The actin density along the actin cables exhibits stronger fluctuations compared to that in PS1, primarily due to the combined effects of fast For3p association and detachment from cable tips. (H) In contrast to PS1, the appearance of For3p dots along the cable body is very rare. In PS2, the experimentally observed For3p dots need to be attributed to some additional mechanism that could occasionally help carry For3p into the cable body.
(0.20 MB PDF)

Figure S4 Summary of simulation results of Parameter Set 3 (Table S2), same plots as in SI Fig. 9 and 10. (A)–(B) Similarly to PS1 and PS2, PS3 generates the same qualitative dependence of the number of For3p dimers per cable tip, average cable length, and average cable retrograde flow rate on the fraction of active actin. (C)–(D) The simulated FRAP curves of For3p at cell tips fit the experimental data. Similarly to PS2, the recovery of For3p at cell tips is dominated by cytoplasmic For3p due to the relatively low fraction of For3p at cable tips and cable body. We used a FRAP region of size 1.6 µm as compared to 1.4 µm. (E)–(F) The cytoplasmic actin concentration exhibits a small concentration gradient. The cytoplasmic concentration of For3p exhibits a significant concentration gradient due to the massive transport of For3p by cable retrograde flow. (G) Similarly to PS2, the actin density along the actin cables exhibits stronger fluctuations as compared to those of PS1. (H) In contrast to PS1 and PS2, a large amount of For3p dimers are associated with the actin cable body. This pattern could be consistent with observations only if the experimental detection sensitivity was ∼6 For3p dimers per pixel.
(0.20 MB PDF)

Figure S5 Results of a model with cortical For3p detachment rate independent of actin polymerization rate. Panels A–C correspond to PS1 (Table 1), PS2 (Table S1), and PS3 (Table S2), respectively. In each case, the rate of For3p detachment was chosen to be the same as the steady rate of cortical For3p detachment in the corresponding model with actin-dependent detachment of the main text (at 100% active actin). The fraction of active actin was then changed, but the rate of detachment remained fixed. Reducing the fraction of active actin has no effect on the number of cortical For3p in PS1 and PS2. In PS3, a cytoplasmic For3p gradient is maintained at steady state by the rapid transport of For3p away from cell tips by actin cables (see Fig. S4H). This gradient disappears in the presence of LatA which depolymerizes the cables, thus allowing more of For3p to associate with the cortical foci at the tips as the fraction of active actin decreases.
(0.23 MB PDF)
