# Supplementary material for: Model of For3p-Mediated Actin Cable Assembly in Fission Yeast
Source: PLoS One. 2008 Dec 31;3(12):e4078. doi: 10.1371/journal.pone.0004078 (PMC2605553; doi:10.1371/journal.pone.0004078)
Supplement: Text S1 — (0.03 MB PDF) [file pone.0004078.s001.pdf]

Supporting Information Text

1. Description of computational model

In this section we provide additional details on the reaction-diffusion scheme of the computational model introduced in section “Model and Assumptions” of the main text. We implemented the processes shown in Fig. 1B-D of the main text as follows (the process numbers in the following items correspond to the numbers in Fig. 1B-D):

1. Actin cable polymerization. The rate of actin polymerization of the ith cable is $\lambda\, k_{\mathrm{A}}^{+} C_{\mathrm{A}}(\mathbf{x}_i) F_{\mathrm{tip}}^{i}$, where $C_{\mathrm{A}}(\mathbf{x}_i)$ is actin cytoplasmic concentration at the tip of the cable at position $\mathbf{x}^i$ and F_{tip}^{i} is the number of For3p dimers bound to that tip. All For3p bound to cable tips are assumed active. Polymerized actin becomes part of the actin mass in the cable.

2. Association of For3p with cable tips. Cytoplasmic For3p at lattice site $\mathbf{x}_i$ (tip of the ith cable) associates with the cortex with rate $\lambda\, k_{\mathrm{F}}^{+} C_{\mathrm{F}}(\mathbf{x}_i)$, where $C_{\mathrm{F}}(\mathbf{x}_i)$ is the local concentration at $\mathbf{x}_i$. We use the Monte Carlo method to calculate the probability of association separately for each For3p dimer.

3. For3p detachment from cell cortex. Similarly to the analytical model, the rate of cortical For3p detachment, r_d, is linearly dependent on the actin polymerization rate per For3p dimer, $r_d = \lambda\, k_{\mathrm{A}}^{+} C_{\mathrm{A}}(\mathbf{x}_i)/p$, and implemented with the Monte Carlo method. After release from the cell cortex, the For3p dimer is passively transported along the cable by retrograde flow.

4. Actin cable retrograde flow. We assume that the speed of retrograde flow, v_{retro}, is limited by the polymerization rate per For3p dimer: $v_{\mathrm{retro}} = k_{\mathrm{A}}^{+} C_{\mathrm{A}}(\mathbf{x}_i)\ell_a$, where $\ell_a = 2.77$ nm is the half length of an actin subunit. We do not account for the possible effects of myosin pulling that may contribute as in budding yeast [1]. Actin and individual For3p dimers are passively transported along the 1D cable lattice according to the time-dependent retrograde flow.

5. Actin cable disassembly. We considered two simple models for actin cable disassembly, as mentioned in the main text: (i) uniform disassembly rate along the cable, and (ii) disassembly rate along the cable which depends on the age of the local actin cable segment. We used a Monte Carlo method to dissociate individual For3p from cables. The disassembled actin and For3p molecules are locally released as functional units to the cytoplasm. In addition, a segment breaks off the cable at any spot whose thickness is thinner than two actin filaments over the length of a 0.02 μm.

6. Actin and For3p diffusion. The diffusion of cytoplasmic actin is solved numerically on the lattice using a diffusion coefficient D_{A}, reflecting boundary conditions at cell edges, and integration time step $\Delta t = 0.001s$. Cytoplasmic For3p dimers perform random walks on the lattice with hopping probability $6D_{\mathrm{F}}\Delta t/\Delta x^2$, where and D_{F} is the diffusion coefficient of For3p dimers.

By reducing the value of Δt to 0.0001s, we checked that the results of the model are independent of the magnitude of the integration step.

2. Stability analysis of the analytical model

In order to explore the stability of the system of Eqs. 1 and 2, we linearized the right-hand sides around the steady state solution and solved for the eigenvalues of the characteristic equations resulting from a 3×3 matrix. For the case $r_{\mathrm{A}}^{-} = r_{\mathrm{F}}^{-}$, the eigenvalues have simple analytical expressions from which one can show that they all have gave negative real parts (one of the three eigenvalues is $-r_{\mathrm{F}}^{-}$ exactly), indicating that the stationary solution of Eq. 3 is a stable solution for the linearized system. For $r_{\mathrm{A}}^{-} \neq r_{\mathrm{F}}^{-}$, we scanned the multi-dimensional space of the model parameters in the region $k_{\mathrm{A}}^{+} \in [0, 100]\mu\mathrm{M}^{-1}\mathrm{s}^{-1}$, $k_{\mathrm{F}}^{+} \in [0, 1000]\mu\mathrm{M}^{-1}\mathrm{s}^{-1}$, $r_{\mathrm{A}}^{-}, r_{\mathrm{F}}^{-} \in [0, 20]\mathrm{s}^{-1}$ and $p \in [0, 5000]$ with other parameters fixed as in Table 1. The cubic characteristic polynomial of the characteristic equations was solved numerically and the real parts of all the eigenvalues were found to be negative, suggesting that the system is stable in this region.

We also examined initial conditions far from the steady state. Eq. 1 and 2 were integrated numerically

with various physical initial conditions (actin/For3p in actin cables and cytoplasm, For3p at cable tips all positive), and we found that the steady state solution always converged to the steady state solution.

References

[1] Huckaba TM, Lipkin T, Pon LA (2006) Roles of type II myosin and a tropomyosin isoform in retrograde actin flow in budding yeast. J Cell Biol 175:957-969.
