# Supplementary material for: Model of For3p-Mediated Actin Cable Assembly in Fission Yeast
Source: PLoS One. 2008 Dec 31;3(12):e4078. doi: 10.1371/journal.pone.0004078 (PMC2605553; doi:10.1371/journal.pone.0004078)
Supplement: Table S1 — (0.02 MB PDF) [file pone.0004078.s002.pdf]

Table S1. Model parameters (Parameter Set 2)

Symbol	Description	Value	Symbol	Description	Value
C_A	Global actin concentration	21 μM [a]	k_A^+	Actin polymerization	2.5 μM^{-1}s^{-1} [d]
F_tot	Total number of For3p dimers	3000 [b]	k_F^+	For3p cortical association	500 μM^{-1}s^{-1} [e]
N_cable	Total number of actin cables	20 [a]	τ_age	Actin filament aging time	5 s [f]
N_patch	Average number of actin patches	50 [a]	$r^-_\mathrm{A,max}$	Aged actin filament disassembly rate	0.1 s^{-1} [f]
D_A	Actin monomer diffusion coefficient	4 μm^{2}s^{-1} [a]	r_A^-	Uniform actin disassembly rate	0.083 s^{-1} [g]
D_F	For3p dimer diffusion coefficient	0.2 μm^{2}s^{-1} [c]	p	For3p processivity	100 [h]
			r_F^-	Uniform For3p disassembly rate	10 s^{-1} [i]

[a] Same as in Table 1.
[b] A slightly larger number of For3p dimers is required to obtain realistic numbers of For3p dimers per cable tip as compared to Parameter Set 1 (Table 1).
[c] A small diffusion coefficient of For3p is required to fit FRAP data, see Fig. S3.
[d] Value reproducing measured cable flow rates.
[e] A large value of k_F^+ is necessary to maintain the required population of For3p at cable tips to balance the fast For3p dissociation from cable tips as assumed.
[f] Value reproducing actin cable lengths and density profiles along actin cables consistent with experiment.
[g] Value for which the analytical model and computational model with age-independent disassambly give identical results in the limit of fast actin and For3p cytoplasmic diffusion coefficients.
[h] Assumption of Parameter Set 2.
[i] Since each For3p dimer at the cable tip polymerizes $\sim$100 actin subunits per second (corresponding to retrograde flow of cables $v_\mathrm{retro} \approx 0.3$ μms^{-1}), the average lifetime of a For3p dimer at a cable tip is $\sim$1 s for $p = 100$. Thus the dissociation rate of For3p from cables needs to be large since otherwise a long stretch of the body of actin cables will be saturated with For3p. For3p is assumed to dissociate from cables with a uniform rate in an actin-independent manner.
