# Supplementary material for: Model of For3p-Mediated Actin Cable Assembly in Fission Yeast
Source: PLoS One. 2008 Dec 31;3(12):e4078. doi: 10.1371/journal.pone.0004078 (PMC2605553; doi:10.1371/journal.pone.0004078)
Supplement: Table S2 — (0.01 MB PDF) [file pone.0004078.s003.pdf]

Table S2. Model parameters (Parameter Set 3)

Symbol	Description	Value	Symbol	Description	Value
C_{A}	Global actin concentration	21 μM [a]	k_{A}^{+}	Actin polymerization	2.5 $\mu\mathrm{M}^{-1}\mathrm{s}^{-1}$ [a]
F_{tot}	Total number of For3p dimers	10000 [b]	k_{F}^{+}	For3p cortical association	500 $\mu\mathrm{M}^{-1}\mathrm{s}^{-1}$ [a]
N_{cable}	Total number of actin cables	20 [a]	τ_{age}	Actin filament aging time	5 s [a]
N_{patch}	Average number of actin patches	50 [a]	$r_{\mathrm{A,max}}^{-}$	Aged actin filament disassembly rate	0.1 s^{-1} [d]
D_{A}	Actin monomer diffusion coefficient	4 $\mu\mathrm{m}^2\mathrm{s}^{-1}$ [a]	r_{A}^{-}	Uniform actin disassembly rate	0.083 s^{-1} [d]
D_{F}	For3p dimer diffusion coefficient	0.2 $\mu\mathrm{m}^2\mathrm{s}^{-1}$ [c]	p	For3p processivity	100 [a]

[a] Values are the same as in Parameter Set 2, Table S1.
[b] In comparison with Parameter Set 2, a slightly larger number of For3p dimers is required to obtain realistic numbers of For3p dimers per cable tip, because a significant fraction of For3p dimers is associated along actin cables.
[c] A small diffusion coefficient of For3p is required to fit FRAP data (see Fig. S4). The requirement for small D_{F} is somewhat less restrictive as compared to Parameter Set 2 since in Parameter Set 3 the large mass of For3p in actin cables also contributes to FRAP recovery.
[d] Value reproducing actin cable lengths and actin cable density profiles along actin cables consistently with experiment. The value of r_{A}^{-} is chosen such that the analytical model and computational model with age-independent disassembly give identical results in the limit of fast cytoplasmic diffusion coefficients. For3p is assumed to dissociate from actin cables with the same rate as actin subunits, see main text.
