# Supplementary figures and images for: Model of For3p-Mediated Actin Cable Assembly in Fission Yeast
Source: PLoS One. 2008 Dec 31;3(12):e4078. doi: 10.1371/journal.pone.0004078 (PMC2605553; doi:10.1371/journal.pone.0004078)

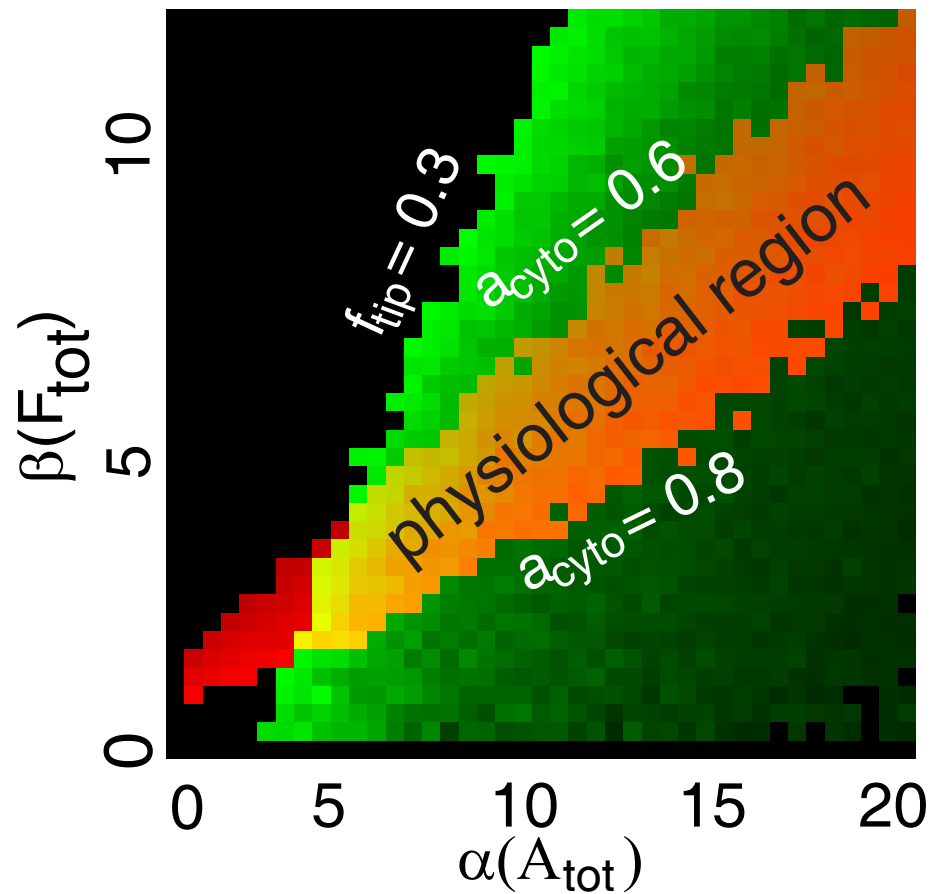

Supplement: Figure S1 — Fraction of For3p at cell tips, f tip, (green) and fraction of cytoplasmic actin, a cyto, (red) as a function of parameters α and β using the computational model. The total concentrations of actin and For3p dimers are varied, with other parameters fixed as in Table 1 (Parameter Set 1). The regions colored red and green show the regions in which 0.6<a cyto<0.8 and 0.05<f tip<0.3, respectively, with the overlapping region in orange. The physiological region is similar to the analytical model (see Fig. 2A in the main text). (0.27 MB PDF) [file pone.0004078.s004.pdf]

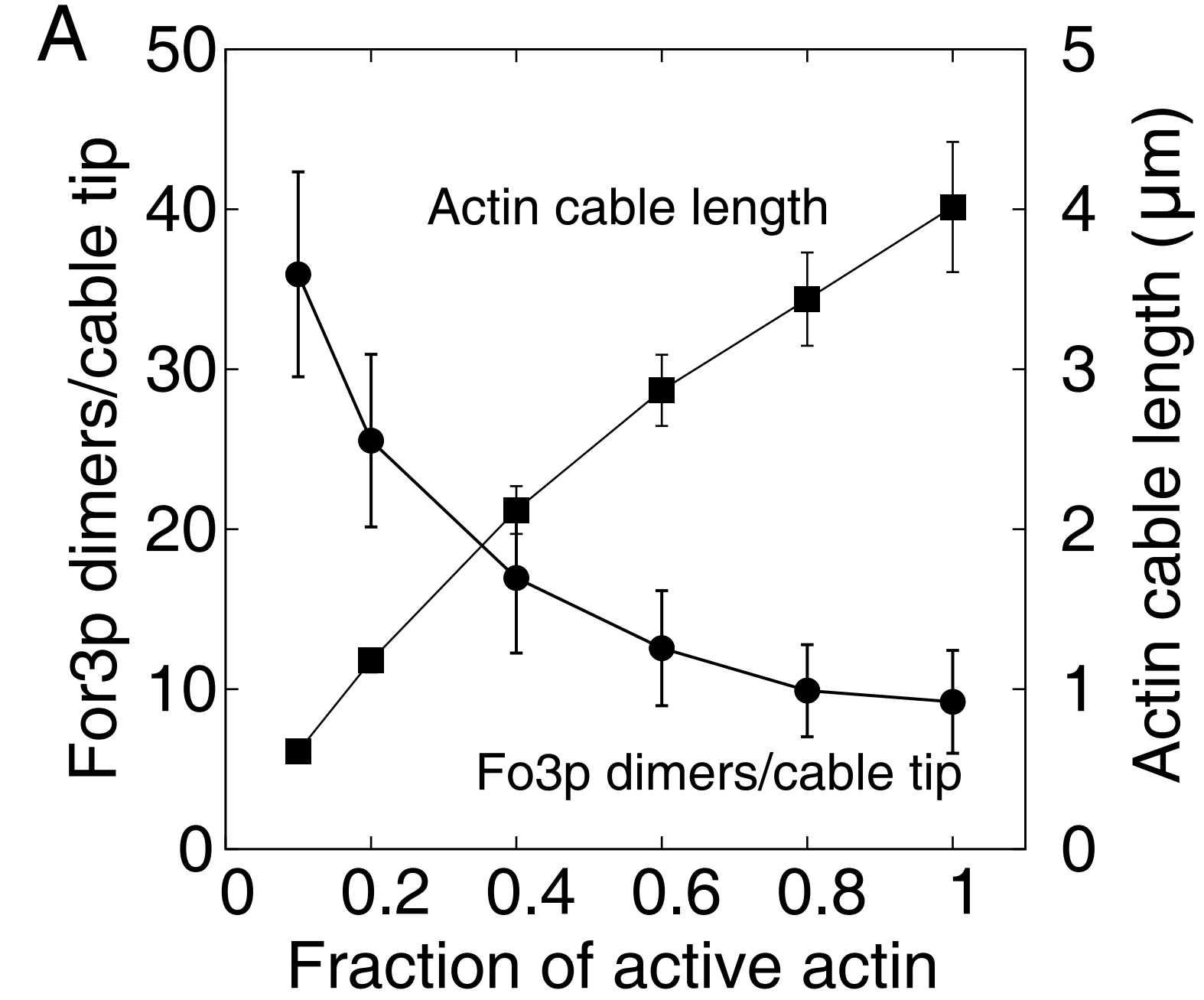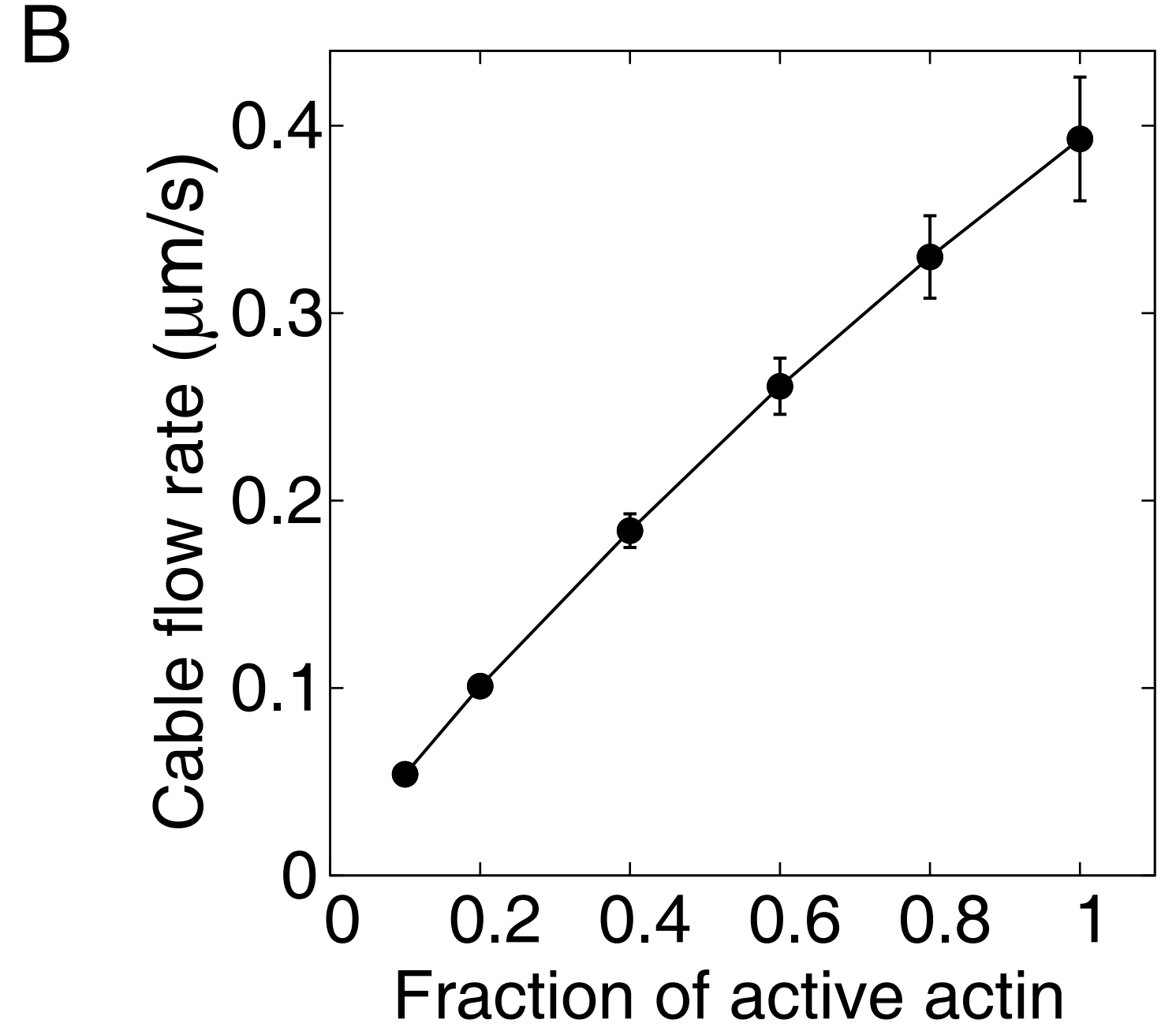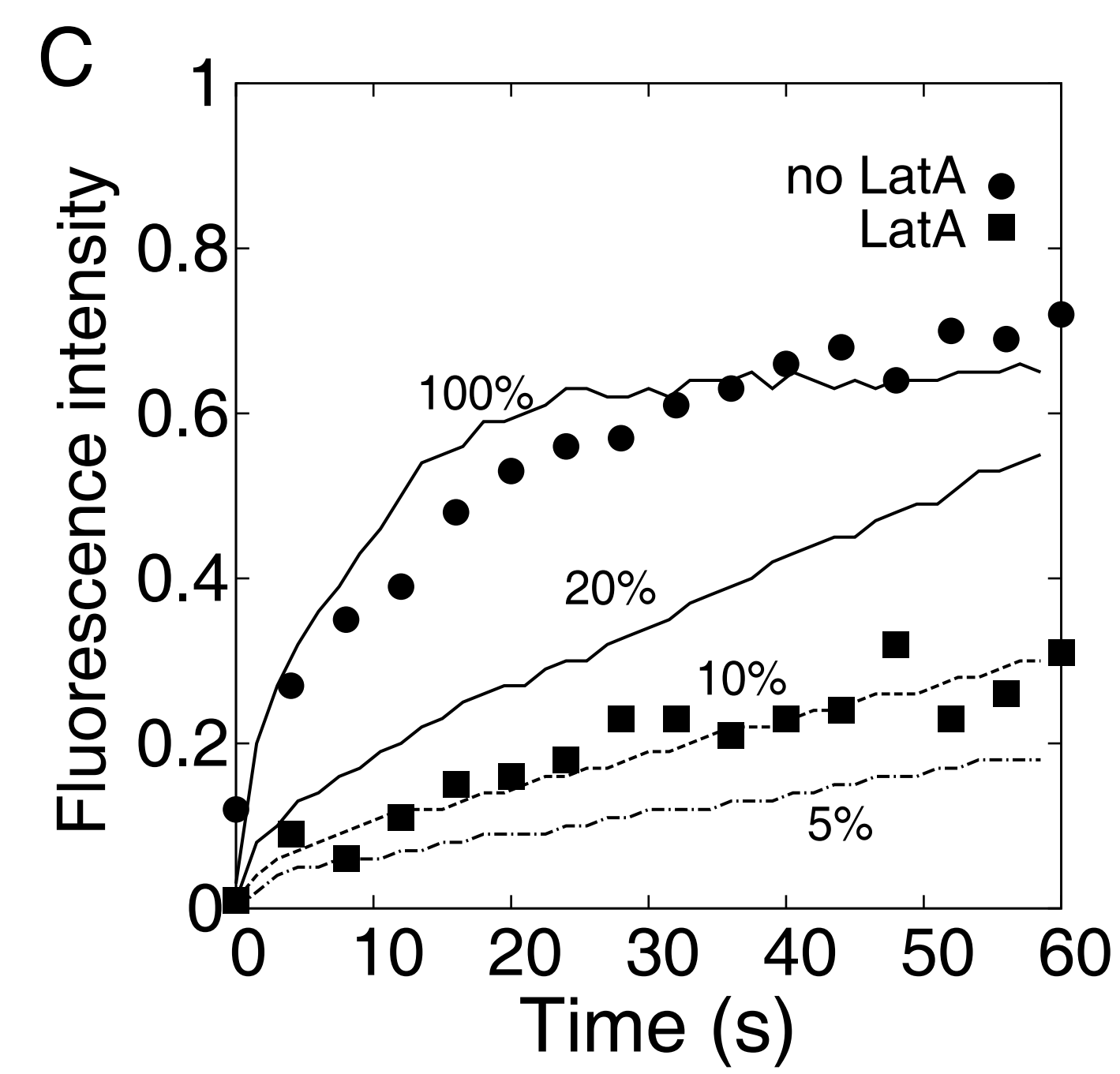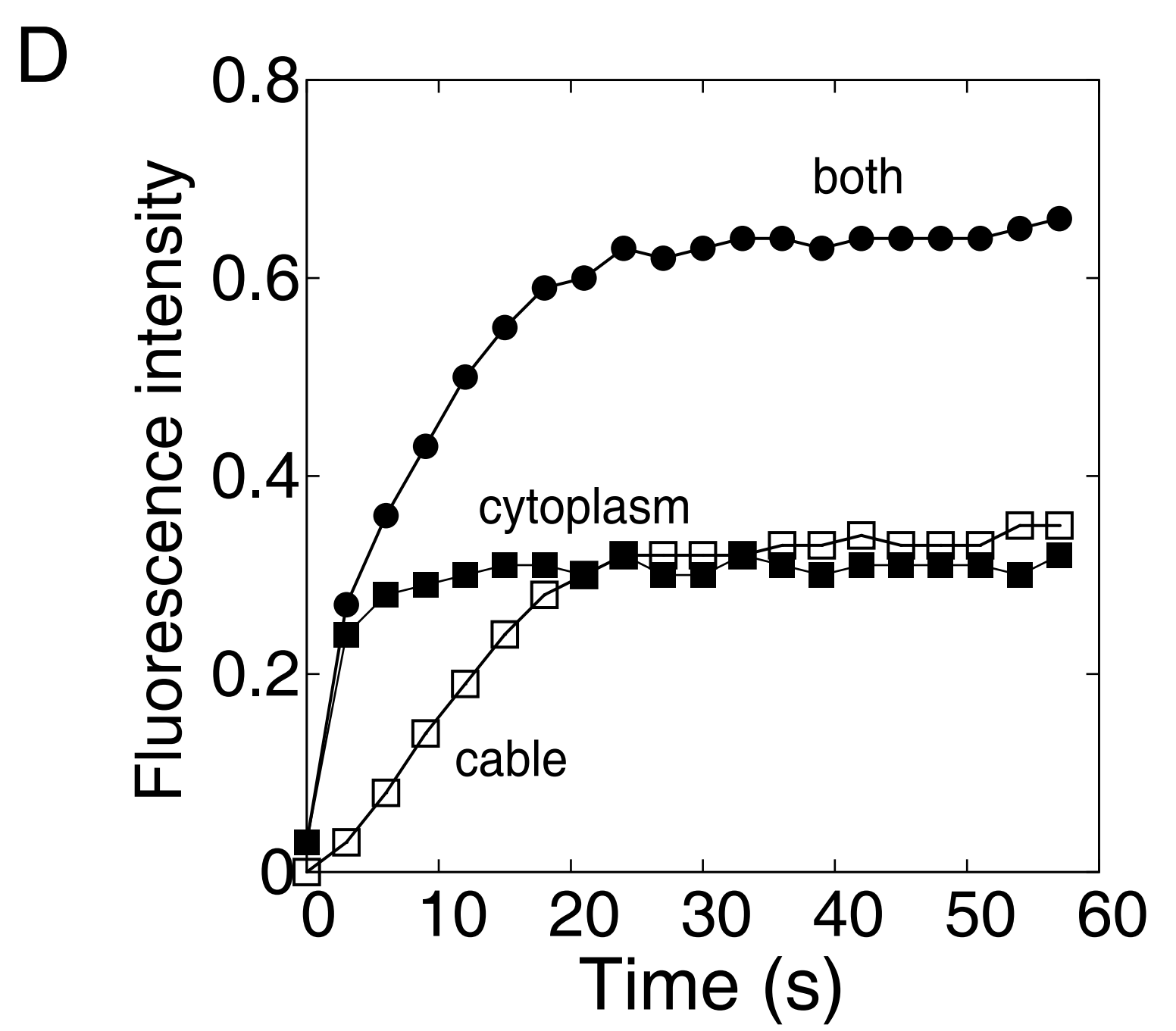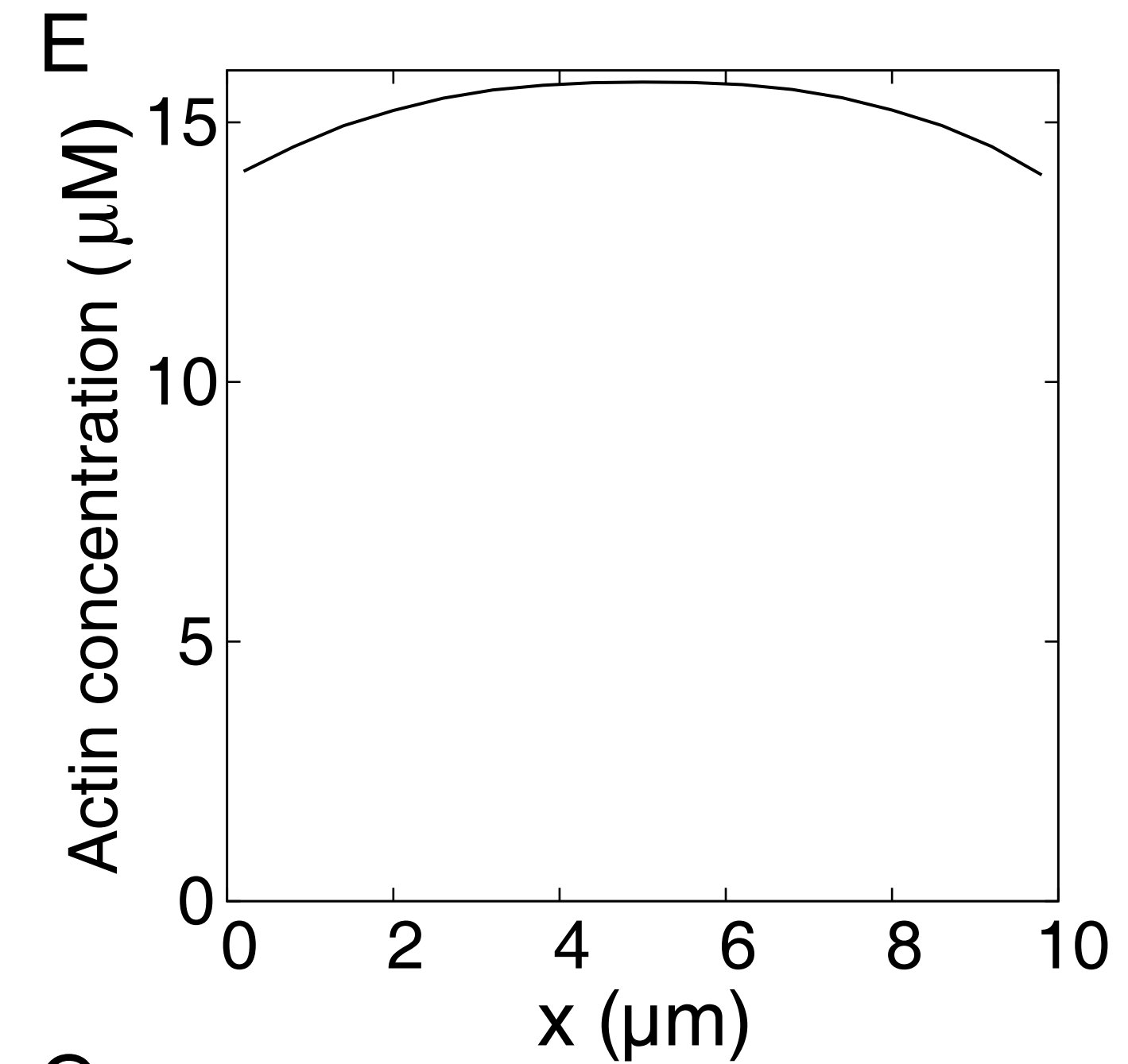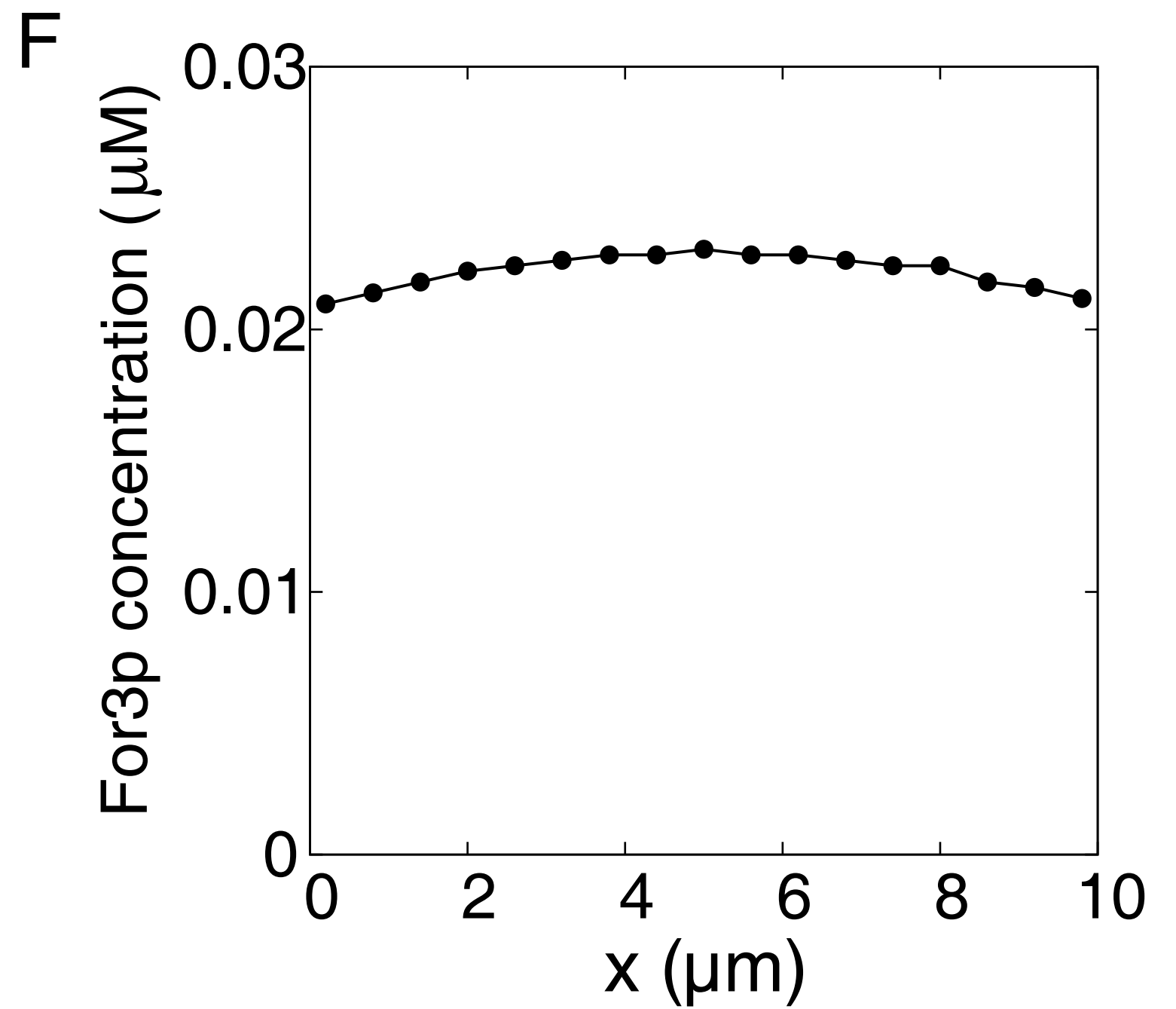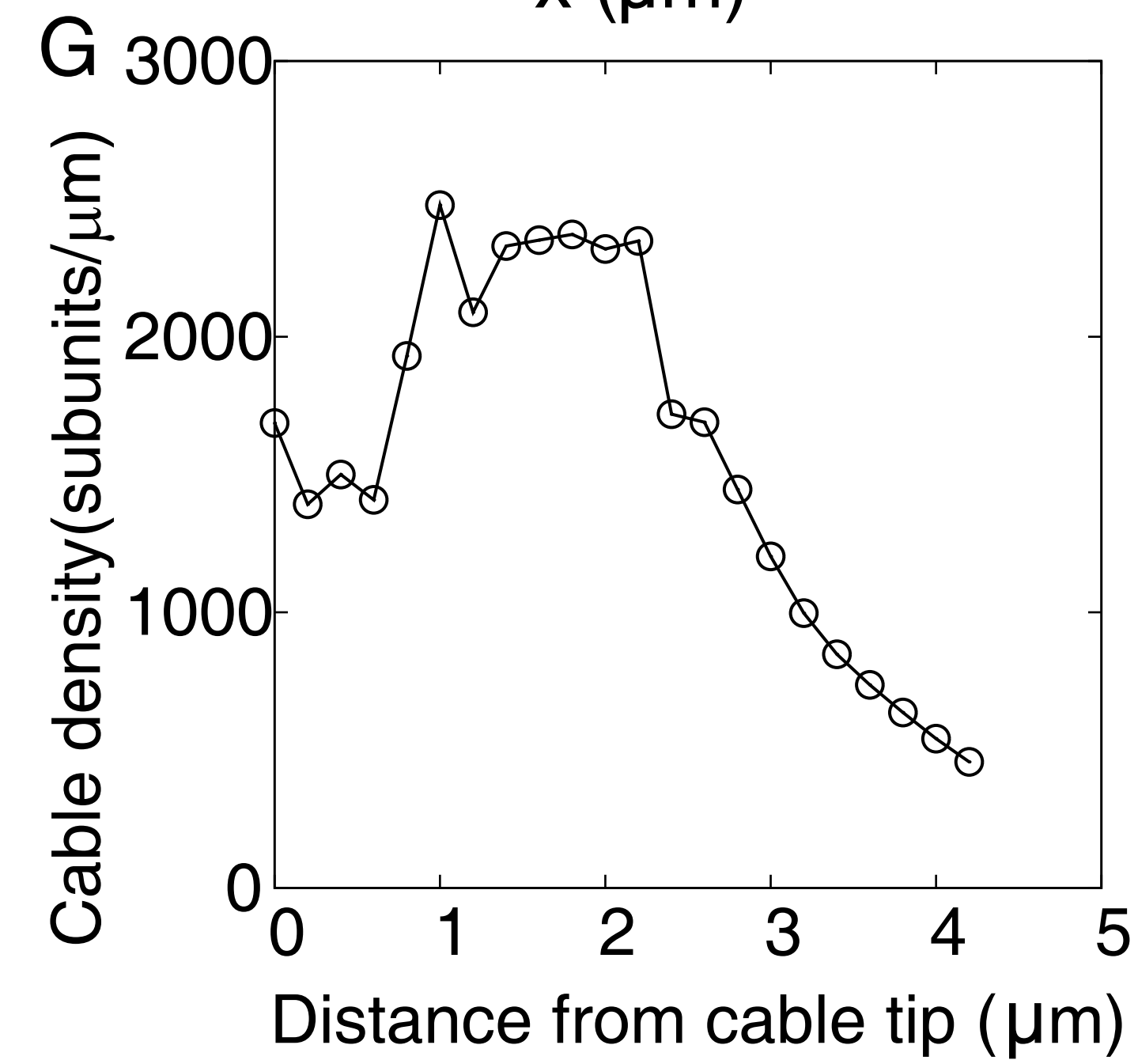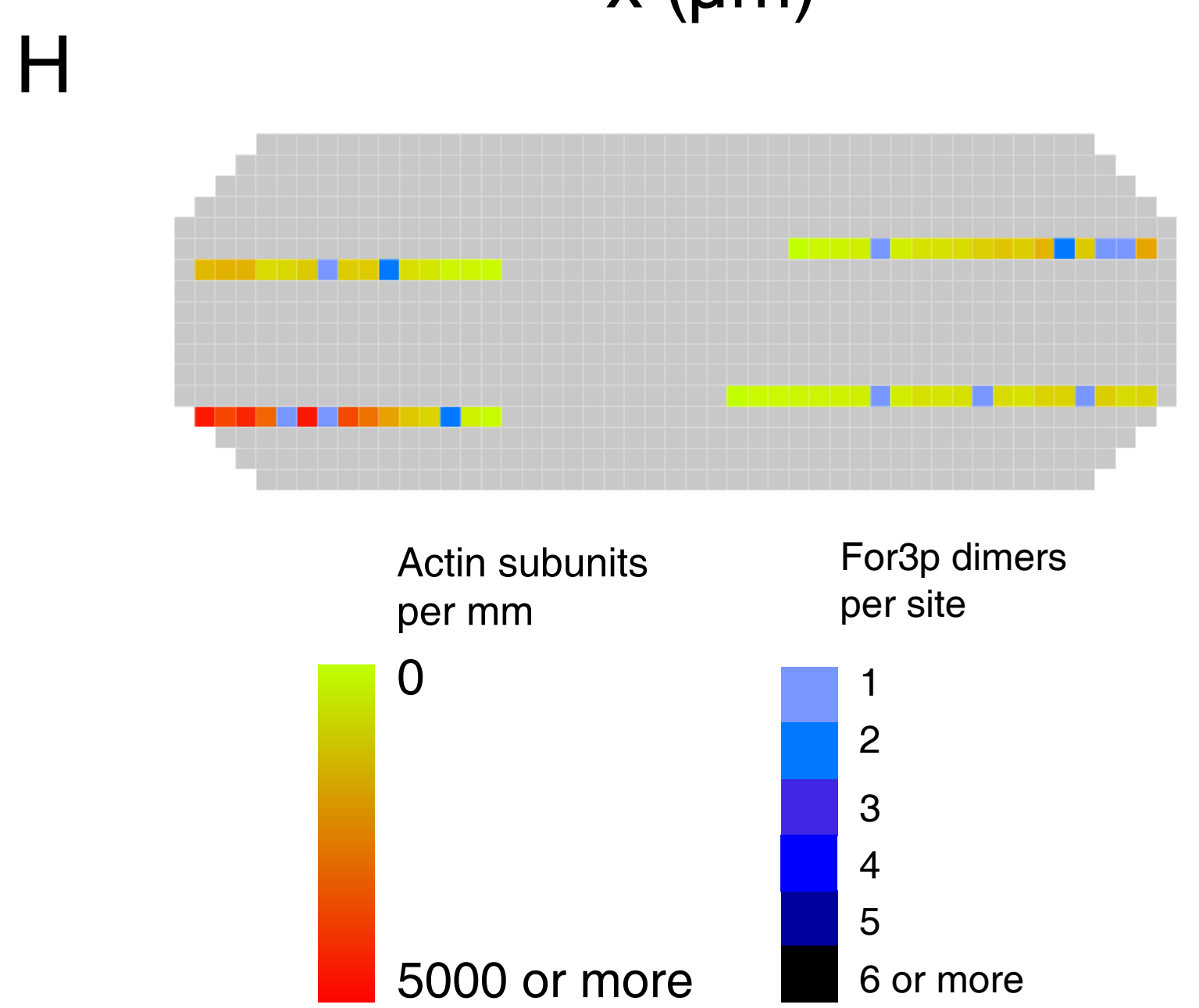

Supplement: Figure S2 — Summary of simulation results of Parameter Set 1 (Table 1). (A)–(B) Dependence of the number of For3p dimers at cable tips, actin cable length, and actin cable flow rate on the fraction of polymerizable actin monomers. The reduction in the fraction of active actin monomers was simulated as a reduction of the polymerization rate constant k + A to mimic the effects of LatA. The trend is consistent with the experiments of Martin and Chang. The error bars show the standard deviation among all actin cables over 100 s. (C)–(D) Simulated FRAP curves of For3p at cell tip, same as Fig. 5. (E)–(F) Cytoplasmic concentration of actin and For3p along the length of the cell, same as Fig. 8. (G) Example of actin filament density along an actin cable, same as the age-dependent curve of Fig. 4B. (H) A snapshot of a 2D slice from the simulation showing the continuum actin field and For3p dots along actin cables. (0.19 MB PDF) [file pone.0004078.s005.pdf]

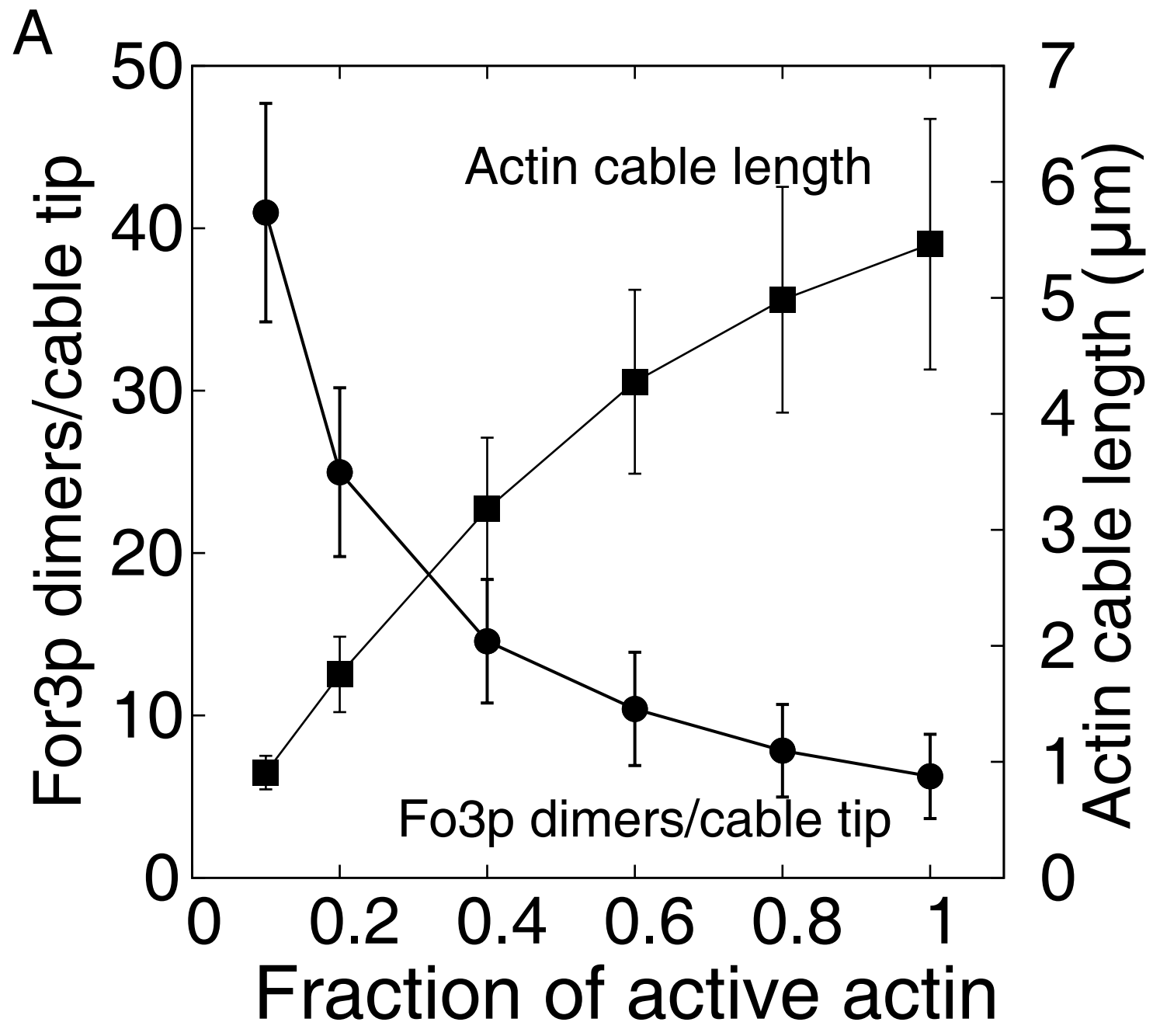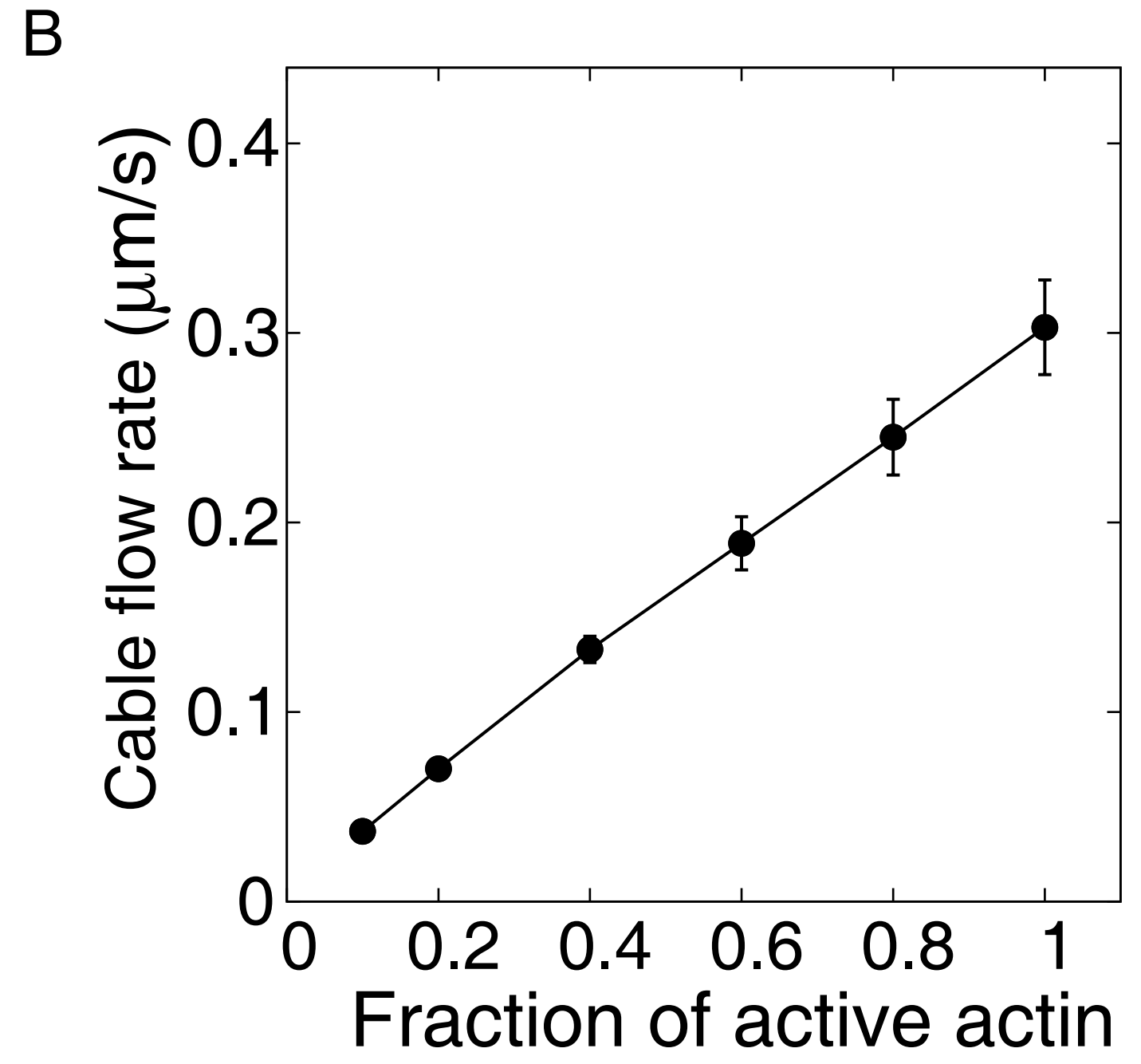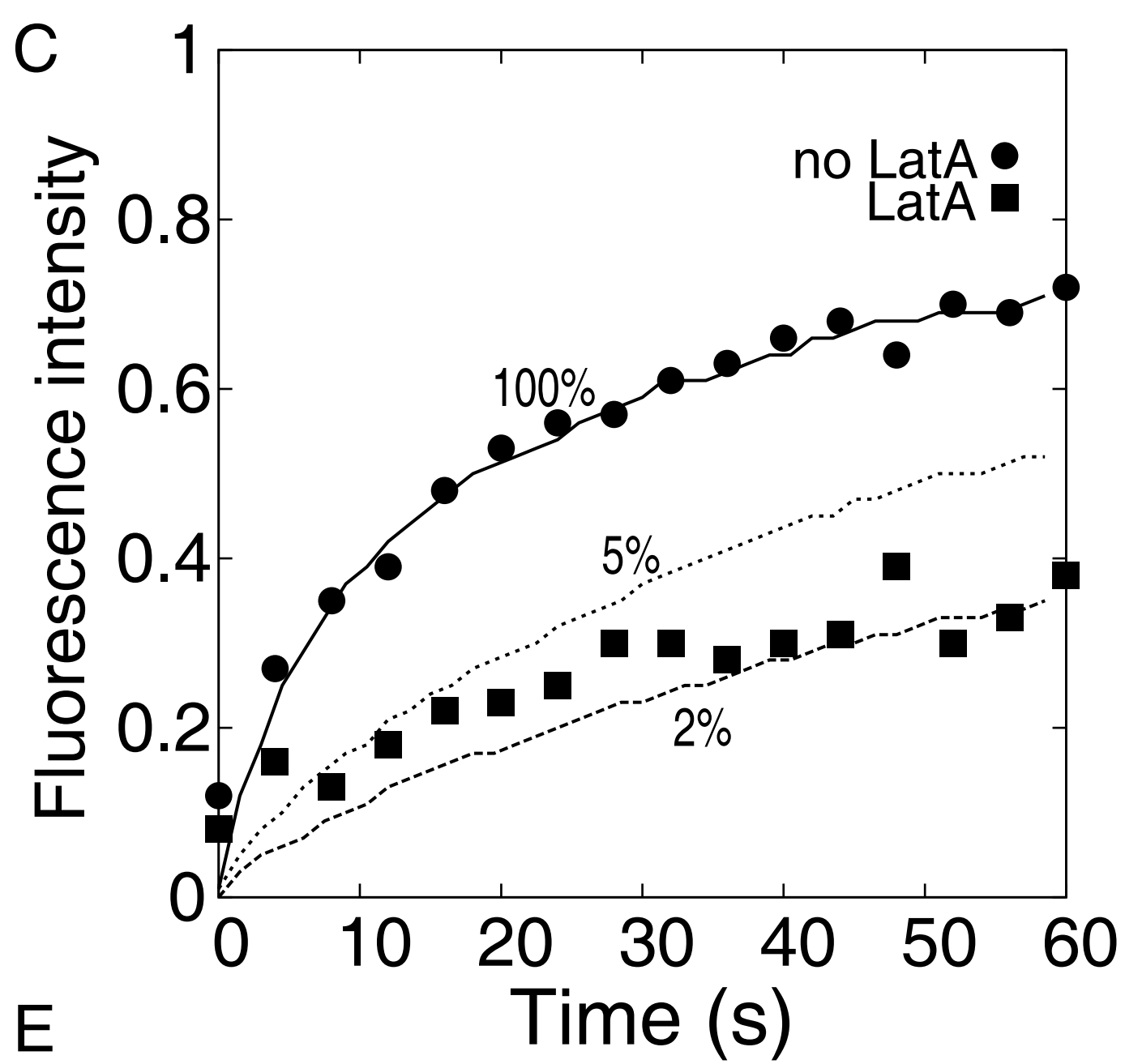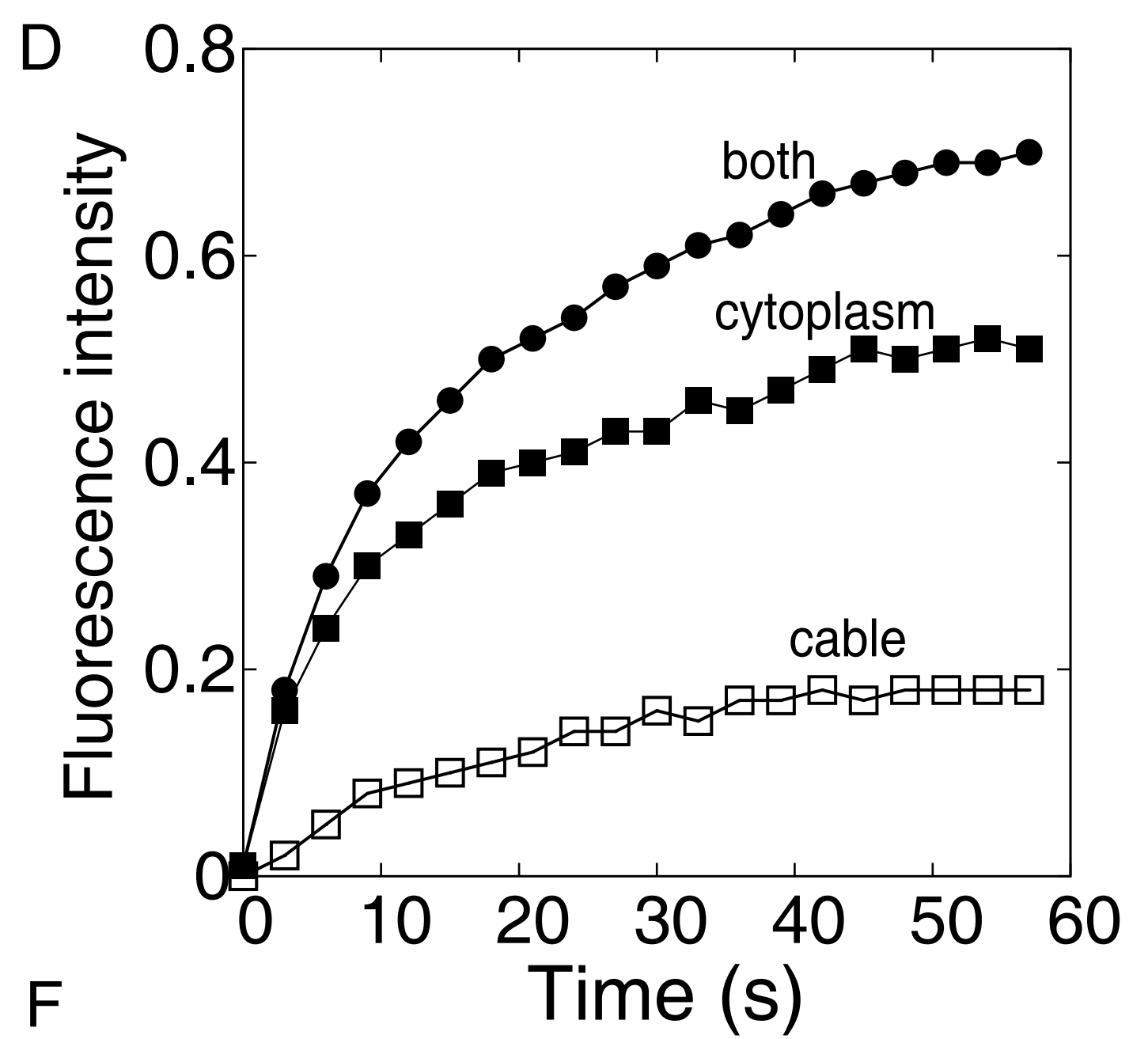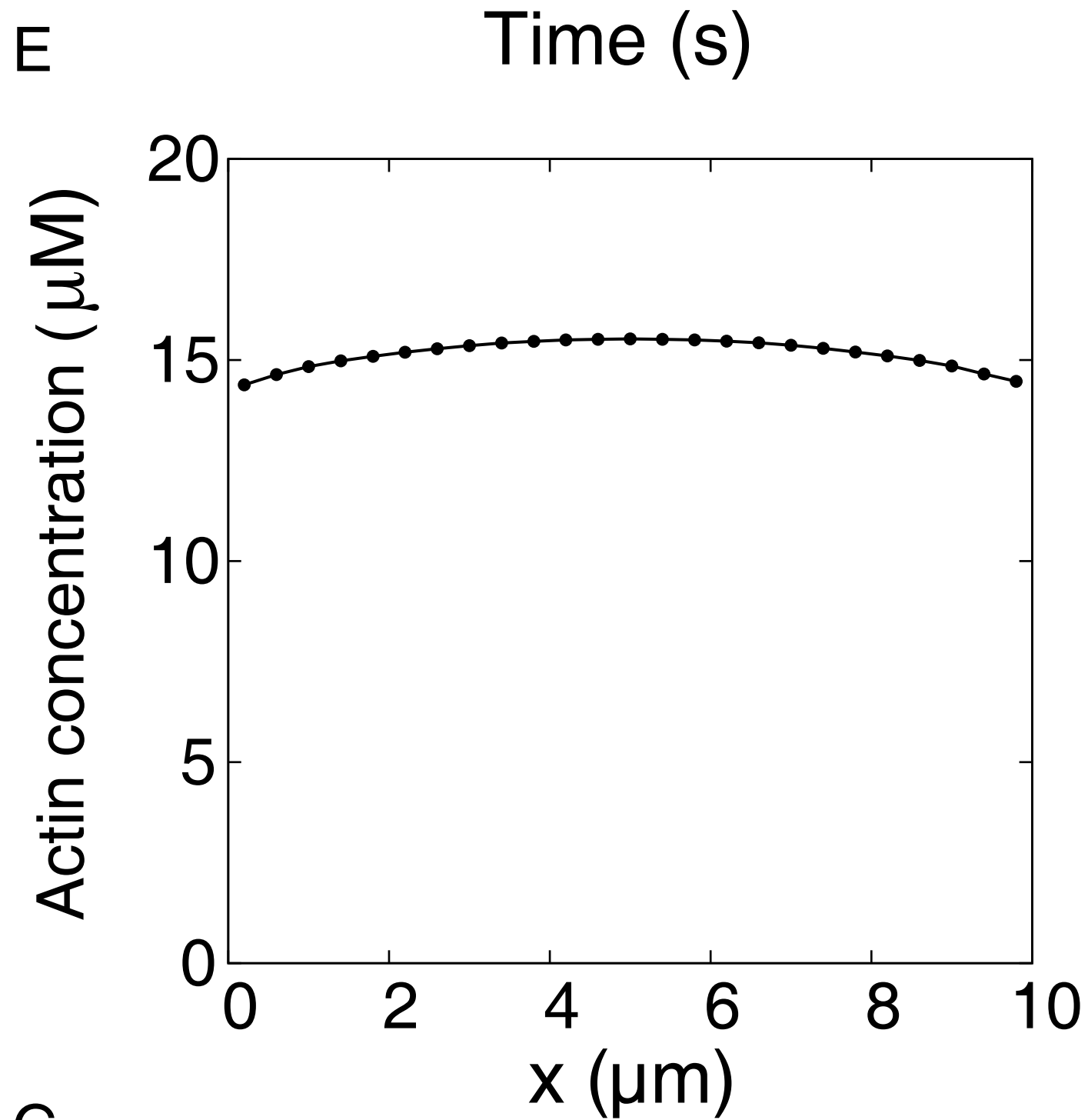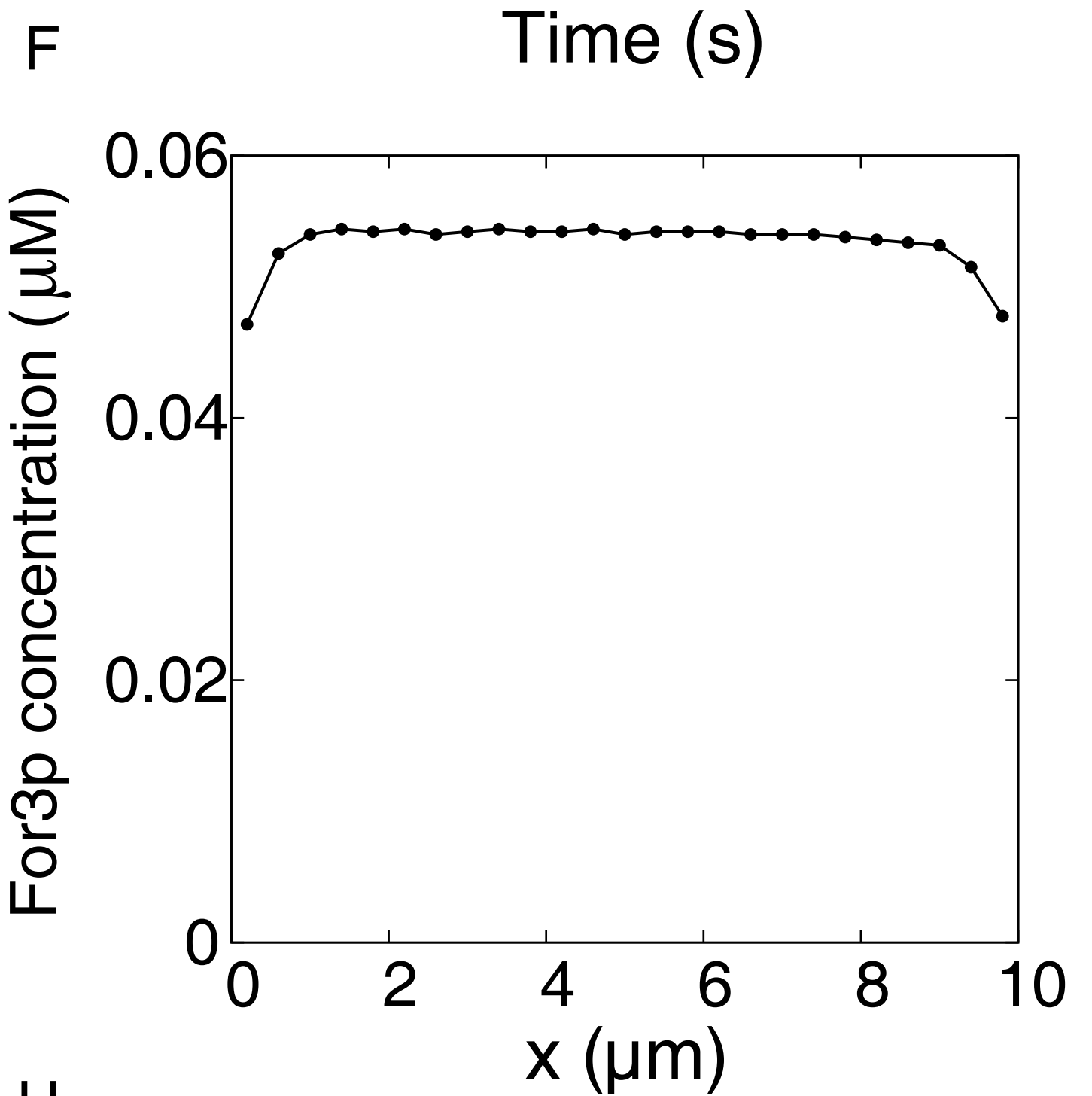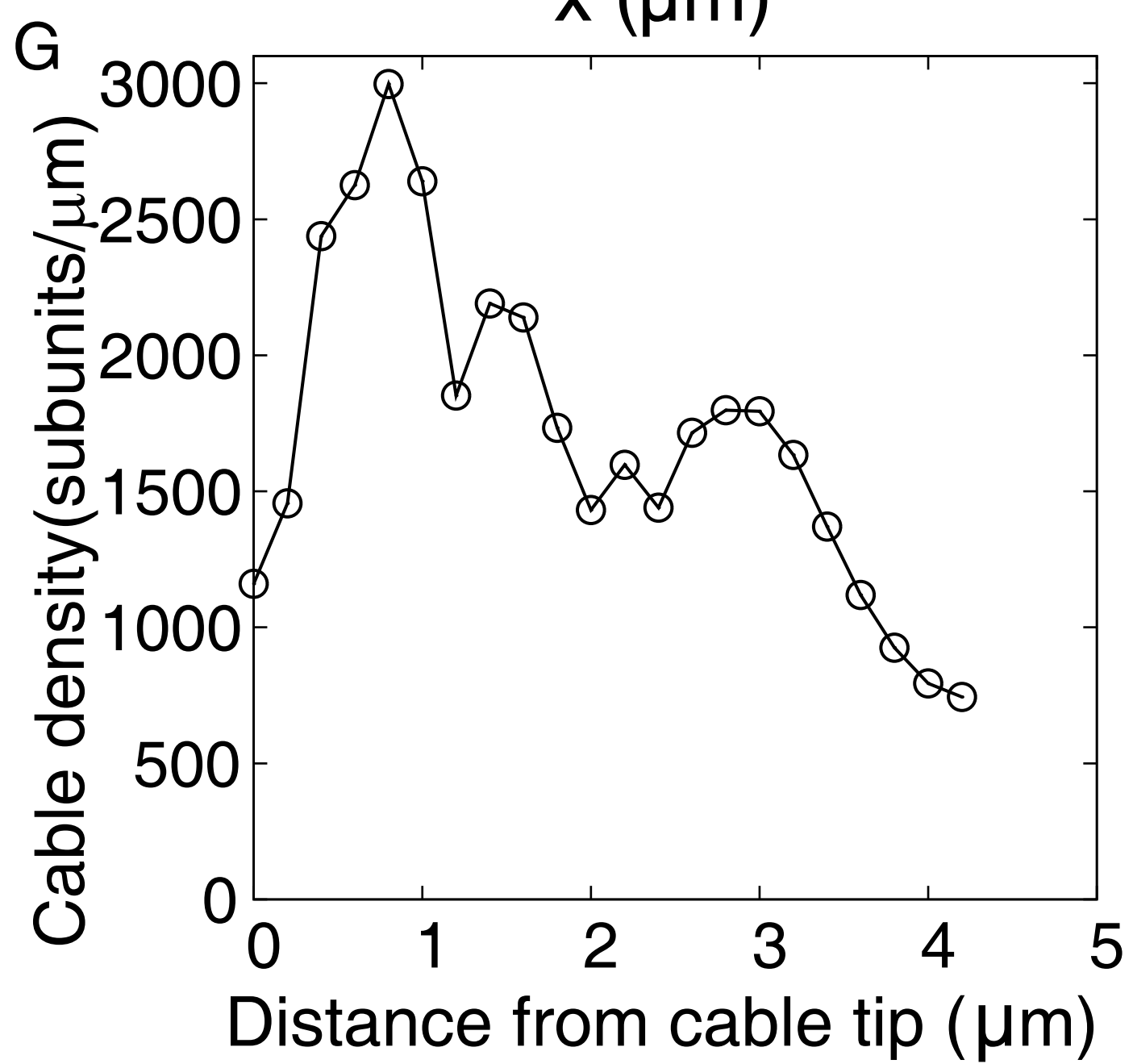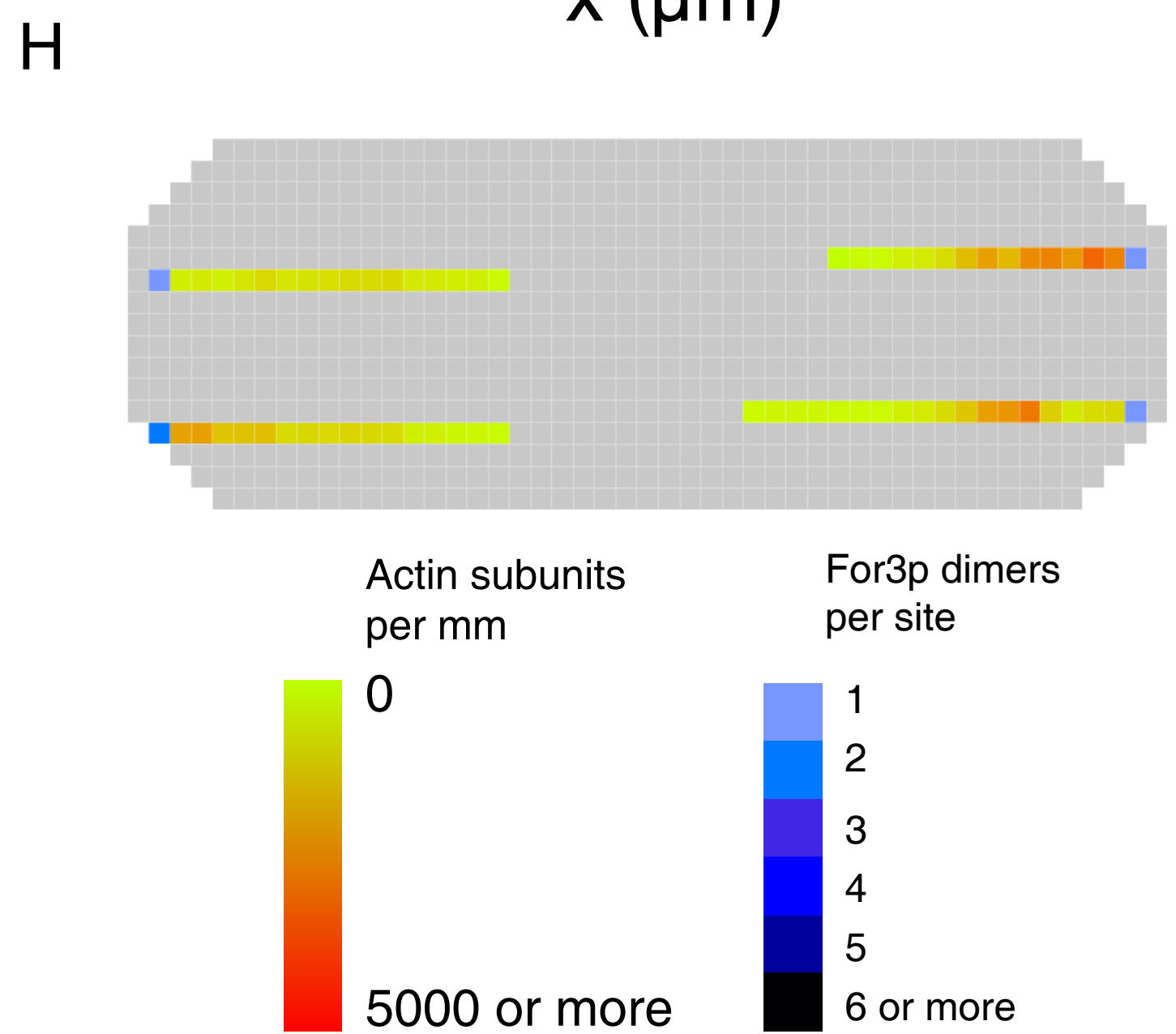

Supplement: Figure S3 — Summary of simulation results of Parameter Set 2 (Table S2), same plots as in Fig. S2. (A)–(B) PS2 exhibits similar trends to PS1 for the number of For3p dimers per cable tip, average cable length, and average cable retrograde flow rate. (C)–(D) The simulated FRAP curves of For3p near the cell tip fit the experimental data in the presence and absence of LatA simulated as a reduction of active cytoplasmic actin (numbers next to curves). Compared to PS1, the recovery of For3p at cell tips is dominated by cytoplasmic For3p due to the relatively low fraction of For3p at cable tips and cable body. (E)–(F) Similarly to PS1, the cytoplasmic concentration of actin exhibits a concentration gradient, and the cytoplasmic concentration of For3p is approximately uniform along the cell. (G) The actin density along the actin cables exhibits stronger fluctuations compared to that in PS1, primarily due to the combined effects of fast For3p association and detachment from cable tips. (H) In contrast to PS1, the appearance of For3p dots along the cable body is very rare. In PS2, the experimentally observed For3p dots need to be attributed to some additional mechanism that could occasionally help carry For3p into the cable body. (0.20 MB PDF) [file pone.0004078.s006.pdf]

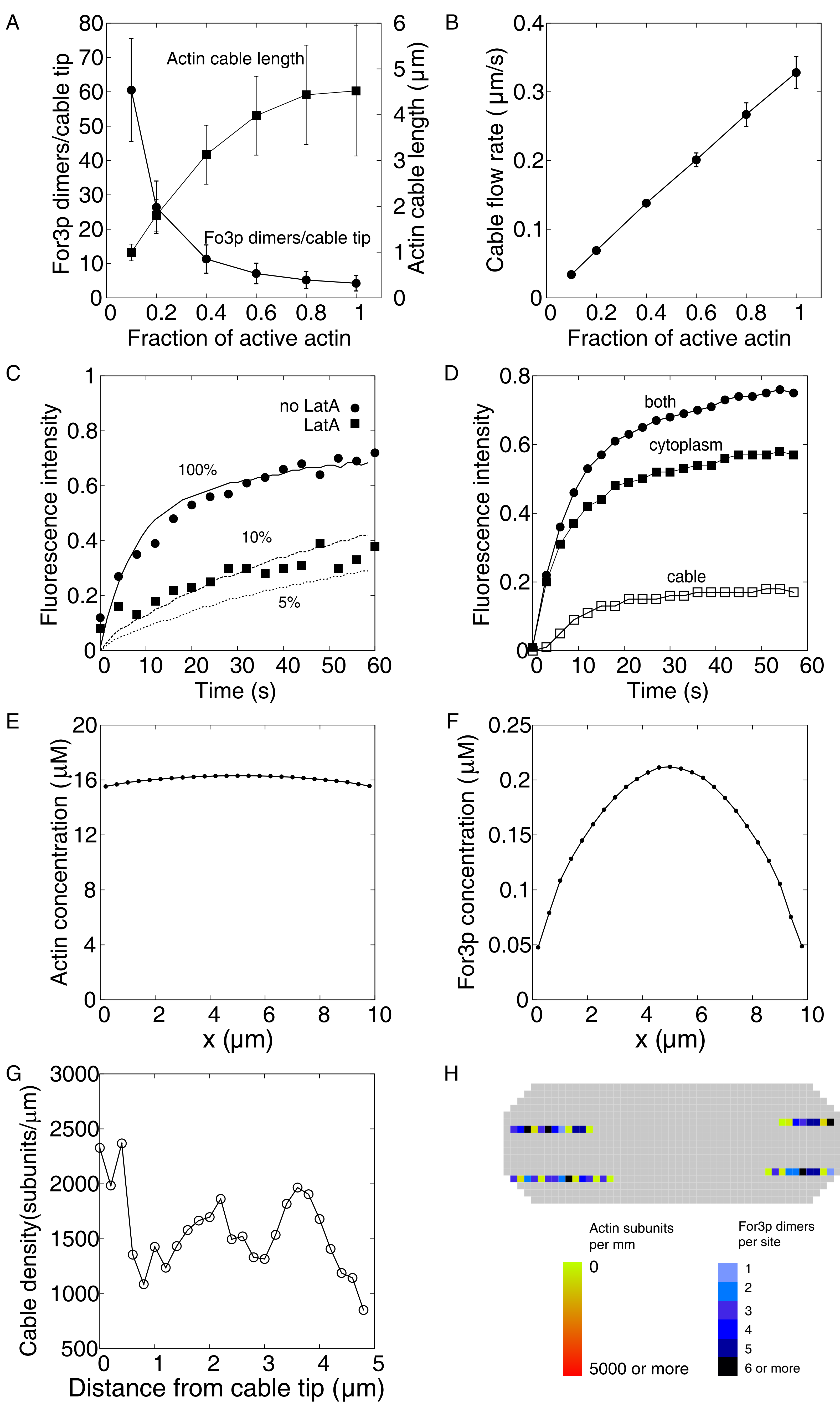

Supplement: Figure S4 — Summary of simulation results of Parameter Set 3 (Table S2), same plots as in SI Fig. 9 and 10. (A)–(B) Similarly to PS1 and PS2, PS3 generates the same qualitative dependence of the number of For3p dimers per cable tip, average cable length, and average cable retrograde flow rate on the fraction of active actin. (C)–(D) The simulated FRAP curves of For3p at cell tips fit the experimental data. Similarly to PS2, the recovery of For3p at cell tips is dominated by cytoplasmic For3p due to the relatively low fraction of For3p at cable tips and cable body. We used a FRAP region of size 1.6 µm as compared to 1.4 µm. (E)–(F) The cytoplasmic actin concentration exhibits a small concentration gradient. The cytoplasmic concentration of For3p exhibits a significant concentration gradient due to the massive transport of For3p by cable retrograde flow. (G) Similarly to PS2, the actin density along the actin cables exhibits stronger fluctuations as compared to those of PS1. (H) In contrast to PS1 and PS2, a large amount of For3p dimers are associated with the actin cable body. This pattern could be consistent with observations only if the experimental detection sensitivity was ∼6 For3p dimers per pixel. (0.20 MB PDF) [file pone.0004078.s007.pdf]

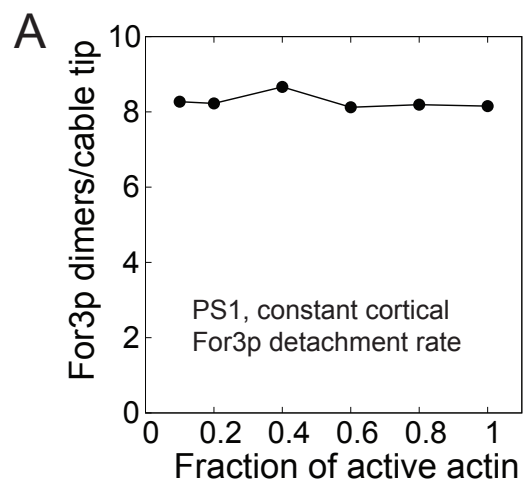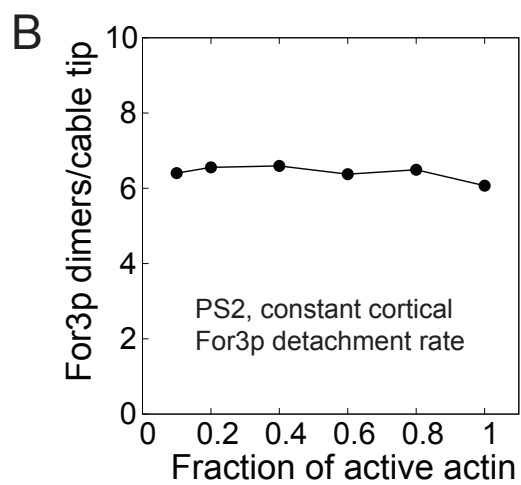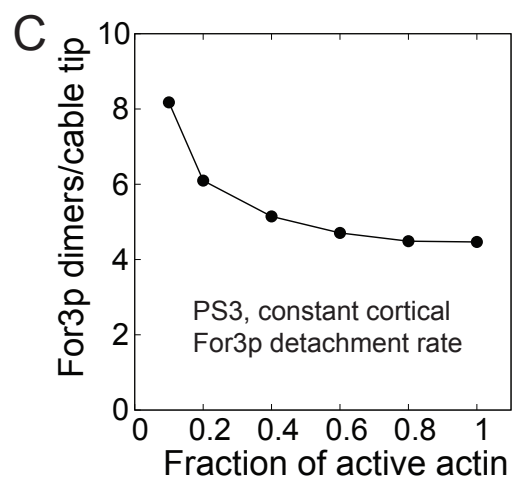

Supplement: Figure S5 — Results of a model with cortical For3p detachment rate independent of actin polymerization rate. Panels A–C correspond to PS1 (Table 1), PS2 (Table S1), and PS3 (Table S2), respectively. In each case, the rate of For3p detachment was chosen to be the same as the steady rate of cortical For3p detachment in the corresponding model with actin-dependent detachment of the main text (at 100% active actin). The fraction of active actin was then changed, but the rate of detachment remained fixed. Reducing the fraction of active actin has no effect on the number of cortical For3p in PS1 and PS2. In PS3, a cytoplasmic For3p gradient is maintained at steady state by the rapid transport of For3p away from cell tips by actin cables (see Fig. S4H). This gradient disappears in the presence of LatA which depolymerizes the cables, thus allowing more of For3p to associate with the cortical foci at the tips as the fraction of active actin decreases. (0.23 MB PDF) [file pone.0004078.s008.pdf]
